# Prevalence of Hepatitis B Virus Infection in Cameroon: A Systematic Review and Meta‐Analysis

**DOI:** 10.1111/jvh.70219

**Published:** 2026-08-05

**Authors:** Iya Maliki, Edward J. D. Webb, Rose Armelle Ada, Bryony Dawkins, Aaron G. Lim

**Affiliations:** ^1^ Academic Unit of Health Economics Leeds Institute of Health Sciences, University of Leeds Leeds UK; ^2^ Department for the Control of Disease, Epidemics and Pandemics Ministry of Public Health Yaoundé Cameroon; ^3^ Population Health Sciences, Bristol Medical School University of Bristol Bristol UK

## Abstract

Chronic hepatitis B virus (HBV) infection is a global public health problem, and many regions of Sub‐Saharan Africa are disproportionately affected, including Cameroon, where prevalence has previously been shown to be high. This study aimed to provide up‐to‐date estimates of HBV prevalence in Cameroon. We searched Medline, Global Health, Embase, Web of Science, Cochrane Library, Africa Index Medicus and the Journal of Health Science and Disease at the University of Yaounde. We included studies reporting HBV surface antigen (HBsAg) prevalence in the general population and subpopulations. A random‐effects meta‐analysis was used to generate pooled estimates of HBsAg prevalence, overall and for population subgroups including those considered at relatively higher‐risk and relatively lower‐risk of HBV infection. Heterogeneity was assessed using the index of heterogeneity (*I* ^*2*^). Quality was assessed using the Joanna Briggs Institute critical appraisal tool. Out of 1236 records, 92 papers with 100 studies were included in the meta‐analysis. Overall prevalence of HBV in Cameroon was 9.98% (95% confidence interval [CI] 9.00–11.02) with high heterogeneity (*I* ^*2*^ *= 98.10%*)*.* The prevalences for relatively lower‐risk and higher‐risk populations were8.94% (95% CI 7.84–10.11) and 12.70% (95% CI 10.28–15.32), respectively. In subgroup analyses, we found that sex, geographical area (regions), population risk groups and study design were the main sources of heterogeneity. Overall, HBV prevalence remains high in Cameroon. Our findings suggest an urgent need for health interventions to halt the spread of the disease, with particular attention needed for males and regions with the highest prevalence.

**Abbreviations**
CI: confidence interval
EIA: enzyme immune assay
ELISA: enzyme‐linked immunosorbent assay
HBV: hepatitis B virus
*I* ^*2*^
: index of heterogeneity statistics
JBI: Joanna Briggs Institute
MoH: Ministry of Health
RDT: rapid diagnostic test

## 1 Introduction

According to the 2024 World Health Organization (WHO) Global Hepatitis Report, nearly 254 million people worldwide live with chronic hepatitis B virus (HBV) [1]. Globally, the number of people newly infected with HBV in 2022 was estimated at about 1.2 million people, and there were about 1.1 million HBV‐related deaths the same year [1]. The majority of new infections (63%) and deaths (86%) from HBV globally occurred in the Sub‐Saharan Africa region, with deaths primarily being caused by complications of the disease (liver cancer and cirrhosis) at the chronic stage [1, 2].

Sub‐Saharan Africa is known as a region with high endemicity, with over 65 million people living with HBV [1, 3]. However, less than 5% of people living with chronic HBV infection in Africa are diagnosed, and among those who are, hardly 5% are receiving treatment [1]. These figures suggest that many people are still unaware of their HBV infection status, have limited knowledge about the condition and its prevention and treatment and/or may not have appropriate treatment options available [1, 3].

The prevalence of chronic HBV in Cameroon has previously been estimated to be 11.9% by the Ministry of Health (MoH), as reported in the National Strategic Plan (2020) developed to fight against viral hepatitis [4]. However, this estimate was produced using data from people aged 15 to 59 years from the Demographic and Health Survey (DHS) 2011 and so is based on outdated data. In addition, there is significant variability across the 10 regions of the country [4], and there is no study reporting the prevalence at country level in all age and sex groups. According to the National Strategic Plan [4], two regions have high prevalence (> 15%: Far‐North and Nord), seven regions have moderate prevalence (8%–15%: Centre, Littoral, South, East, Southwest, Adamawa and West) and one region has low prevalence (< 8%: Southwest) (EDS2021) [4].

A systematic review and meta‐analysis published by Bigna et al. in 2017 reported a pooled chronic HBV prevalence of 11.2% in Cameroon [5]. This is slightly lower than the estimated value from the National Strategic Plan of the MoH [4]. Since these prevalence estimates were produced, there have been policy and intervention developments in Cameroon targeting HBV. For example, new screening campaigns have been launched, disease awareness activities have been implemented in communities, and the cost of HBV treatment has been reduced by the government [6]. Such activities aim to reduce HBV prevalence, and so previous estimates may no longer truly reflect the current prevalence. Considering the global prioritization of hepatitis B and the push to eliminate chronic HBV as a public health problem, as in the WHO Global Health Sector Strategy [7], there are likely to be many new studies emerging since previous estimates were produced. As such, a more up‐to‐date meta‐analysis incorporating these new studies into our overall prevalence estimates is required. This will also allow us to explore whether global trends showing reductions in HBV prevalence are also being observed in Cameroon [8]. Consequently, we aim to produce an estimate of HBV prevalence in Cameroon using the most up‐to‐date and best available data from published studies, which can more effectively inform policy and targeted intervention.

## 2 Methods

The protocol for this systematic review and meta‐analysis has been registered in PROSPERO, registration number CRD42025636337, and it is available through this link https://www.crd.york.ac.uk/PROSPERO/view/CRD42025636337.

### 2.1 Search Strategy

A search strategy based on the review by Bigna et al. [5] was developed to identify relevant primary studies reporting the prevalence of hepatitis B surface antigen (HBsAg) across different population groups in Cameroon (see Table S1). A comprehensive search strategy was designed using subject headings and keywords combined using Boolean operators. The search strategy was refined with input from an expert information specialist before adapting it to each database. We searched Ovid MEDLINE, Embase, Global Health, Cochrane Library, Web of Science and the WHO African Index Medicus. In addition, we manually searched for relevant studies in Health Sciences and Disease, which is the *Journal of Faculty of Medicine and Biomedical Sciences* published by the University of Yaounde, Cameroon. We also searched reports on the prevalence of HBV from the National AIDS Control Committee, Cameroon.

All searches were performed without language restriction to identify relevant articles published between 1 January 2000 and 10 February 2025 (when searches were undertaken). Articles published from 1 January 2000 were included as this represents 5 years before the hepatitis B infant vaccine dose was introduced in Cameroon in 2005 and so allows comparison across timeframes linked to the introduction of important HBV‐related policies. In addition, this was the same publication cut‐off used in the previous systematic review and keeping this consistent allows comparison of the impact of new data on the overall prevalence estimates [5]. The search strategy is provided as a supplementary file (see Table S1).

### 2.2 Classification of Relatively Lower‐Risk and Higher‐Risk Groups

Although this study aims to generate an updated estimate of overall prevalence of HBV in Cameroon, it is important to note that there can be variations in risk of HBV infection for different population groups according to their characteristics, and that in turn these differences in risk can result in observed variations in prevalence. Besides estimating overall prevalence, we define two additional population subgroups: (i) Those who are at relatively lower‐risk of being infected with HBV, which includes the general population, pregnant women and blood donors; and (ii) those who are at relatively higher‐risk of being infected because of their potentially heightened exposure to HBV, which includes healthcare workers, people living with HIV infection, pregnant women living with HIV, haemodialysis patients, sickle cell disease patients, liver cancer patients, other cancer patients, people living with disabilities, refugees and prisoners. Note that throughout this article, the terms ‘higher‐risk’ and ‘lower‐risk’ are used to refer to these groups but are not meant to imply a causal link between group characteristics and HBV infection, only that relatively higher or lower prevalences of chronic HBV infection are expected in these groups due to their exposure risks.

### 2.3 Eligibility Criteria

All observational studies (cross‐sectional, cohort and case‐control) reporting chronic HBV prevalence (defined as testing positive for HBsAg) in Cameroon in any language were included among the general population and other subpopulations with lower‐risk and higher‐risk of HBV infection (see definitions above). We excluded studies performed in animals, clinical trials, author‐reply articles, case reports, qualitative studies, intervention studies, systematic reviews, editorials, commentaries, letters to the editor and studies where the full text was not available and sufficient data were not available in the abstract to determine eligibility (see Table S2).

### 2.4 Study Selection

All articles were retrieved and exported first to EndNote 21.1 for deduplication, then Rayyan was used for title and abstract screening [9]. Titles and abstracts were double‐screened independently by two reviewers (I.M. and R.A.A.) to select relevant papers against the eligibility criteria. Dual full‐text screening in Rayyan was then performed, with conflicts being resolved by discussions among reviewers. The PRISMA flow chart was used to present the flow of inclusion and exclusion of studies throughout the study selection process as well as the Centre for Reviews and Dissemination guidelines [10, 11].

### 2.5 Data Extraction

A data extraction template was developed and piloted with the first 10 papers before proceeding to full data extraction. Data extracted included the following: general study and demographic information (authors, year of publication, age, regions, sites of study, settings, cases, sample size and screening tools used), study design, population groups, HBsAg prevalence and information used to extract the numerator and denominator (cases and sample size). Data extraction was completed by I.M. and was checked by a second reviewer (R.A.A.).

### 2.6 Quality Assessment

The Joanna Briggs Institute (JBI) critical appraisal tool for prevalence studies with nine questions to evaluate the validity (internal and external) of a study was used for quality assessment [12]. Each question has four possible answers: Yes, No, Unclear and Not applicable (see Table S3). We defined poor, moderate and high quality as zero to four, five to six and seven to nine yes responses, respectively. These thresholds were chosen due to their use in a recent study by Kassa et al. [13], which uses a similar methodology to this study to estimate viral hepatitis prevalence (for hepatitis C virus rather than HBV) across Sub‐Saharan African countries considered comparable to Cameroon. The utility of the thresholds in a similar disease and context has thus been previously established.

### 2.7 Data Analysis/Synthesis

#### 2.7.1 Overall Estimate of HBV Prevalence

The *metaprop* Stata command was used to compute the overall pooled prevalence of HBV infection [14]. We used random‐effects meta‐analysis to generate estimates of prevalence and index of heterogeneity (*I* ^*2*^) to assess heterogeneity among studies. The estimated pooled prevalence was plotted and visualized using a forest plot. We used Stata 18.0 software for all analyses [15].

#### 2.7.2 Sensitivity Analyses

Several sensitivity analyses were undertaken to explore the impact of inclusion/exclusion of studies with certain characteristics from the analysis. Each sensitivity analysis used random‐effects meta‐analysis to estimate HBV prevalence after we had excluded relevant studies.

First, a sensitivity analysis was undertaken based on the quality assessment results where studies with poor and moderate risk of bias for quality assessment were excluded. An additional sensitivity analysis was undertaken in which studies that did not include ELISA were excluded, as ELISA is considered to be the ‘gold standard’ screening method used. Lastly, a sensitivity analysis was also undertaken considering only population‐based studies after we have excluded studies carried out in hospitals and in prisons, as population‐based studies may be relatively less biased in terms of the exposure for participants.

#### 2.7.3 HBV Prevalence for Population Subgroups

Estimates of HBV prevalence by population group were generated after stabilizing the variance of each study using the Freeman‐Tukey Double Arcsine Transformation [16, 17]. We explored the following in subgroup analyses: HBV risk group, sex, age, study time period, regions, settings (rural/urban) and study design. In addition, each of the subgroup analyses was undertaken separately for lower risk groups and higher risk groups to explore whether the trends observed are consistent.

Data were grouped for subgroup analyses to explore the variation of prevalence across specific groups within a study population. For the subgroup analysis by HBV risk group, estimates of prevalence were generated for relatively lower‐risk groups and relatively higher‐risk groups (see definitions in 2.2). Data were also analysed for two age groups: ≤ 15 years and > 15 years, as these were the most common age group categories reported across the studies (see Table S4 for explanation of how studies were classified into these age groups). We also undertook a subgroup analysis by time period of the study as we expected there may be lower HBV prevalence in recent studies compared to older ones. We grouped studies into those from before the HBV vaccine was introduced in Cameroon (≤ 2005), those from between the vaccine's introduction and the end year of searches of Bigna et al.'s systematic review and meta‐analysis (2006–2016) [5] and studies published after (≥ 2017). Heterogeneity was assessed via forest plots and by using the Cochrane *Q* test, while the *I* ^*2*^ statistic was used to quantify the level of heterogeneity [18, 19, 20, 21].

## 3 Results

### 3.1 Study Selection

**FIGURE 1:**
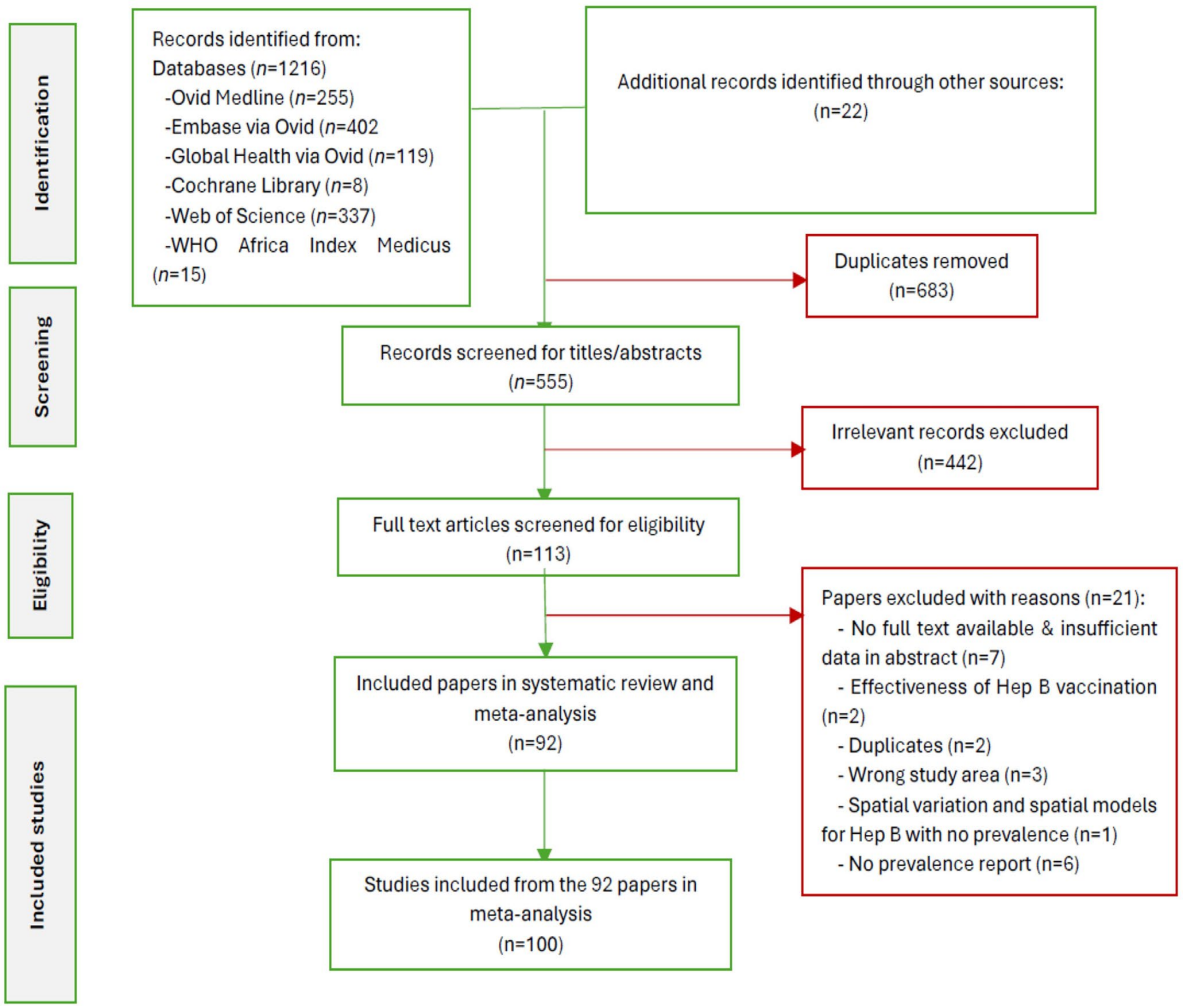
PRISMA diagram—Process of identification and selection of studies including database searches and other sources.

A total of 1238 records were identified by our search strategy. After deduplication, 555 records were eligible for title and abstract screening, of which 442 were excluded because of irrelevance. There were 113 papers full text screened, of which 21 were excluded. Overall, 92 papers with 100 studies were included in the meta‐analysis (see Figure 1). The list of all included studies is available in Table S5.

### 3.2 Characteristics of Included Studies

Table 1 presents the characteristics of all included studies carried out among the general population [22, 23, 24, 25, 26, 27, 28, 29, 30, 31, 32, 33, 34, 35, 36, 37, 38, 39, 40, 41, 42, 43, 44, 45, 46], blood donors [47, 48, 49, 50, 51, 52, 53, 54, 55, 56, 57, 58, 59, 60, 61, 62, 63, 64, 65, 66], pregnant women [3, 55, 57, 67, 68, 69, 70, 71, 72, 73, 74, 75, 76, 77, 78, 79, 80, 81, 82, 83], healthcare workers [55, 84, 85, 86, 87, 88], people living with HIV infection [52, 55, 89, 90, 91, 92, 93, 94, 95, 96, 97, 98, 99, 100, 101], pregnant women living with HIV [67, 102], haemodialysis patients [103, 104, 105], sickle cell disease patients [106], liver cancer patients [25, 29], people with any other type of cancer [107, 108], people living with disabilities [109], refugees [110] and prisoners [111]. The 92 included papers were published from 2001 to 2025 (no included studies published in 2000), with studies carried out from 1994 to 2024 across all 10 regions in Cameroon. The age of participants ranged from 3 months to 101 years old, with a total of 212,167 individuals.

**TABLE 1:** Characteristics of included studies.

Characteristics of included studies	*N* = 100 studies	Percentage* (%)
Population of study		
General population	26	26
Blood donors	21	21
Pregnant women	20	20
Healthcare workers	6	6
HIV‐infected	14	14
HIV‐pregnant women	2	2
Haemodialysis patients	3	3
Sickle cell disease patients	1	1
Liver cancer patients	2	2
Cancer patients (not liver)	2	2
Disabled people	1	1
Refugees	1	1
Prisoners	1	1
Study design		
Case‐control	4	4
Cross‐sectional	91	91
Retrospective cross‐sectional	5	5
Sites of study		
Hospital‐based	86	87.8
Population‐based	10	10.2
Prison‐based	1	1
Hospital‐based and School	1	1
Not reported	2	—
Settings		
Urban area	42	42.4
Rural area	23	23.2
Urban and Rural	34	34.4
Not reported	1	—
Sex		
Male	19	19.3
Female	32	32.7
Male and Female	47	48
Not reported	2	—
Screening tools		
RDT	44	49.4
EIA	12	13.5
ELISA	25	28.1
RDT and ELISA	7	7.9
RDT and EIA	1	1.1
Not reported	11	—

* Percentages do not include ‘not reported’.

### 3.3 Methodological Quality of Studies

Using the JBI tool for quality assessment, 35 studies were classified as poor quality, 46 as moderate quality and 19 as high quality (see Figure S3).

### 3.4 Overall HBV Prevalence From the Random‐Effects Meta‐Analysis

**FIGURE 2:**
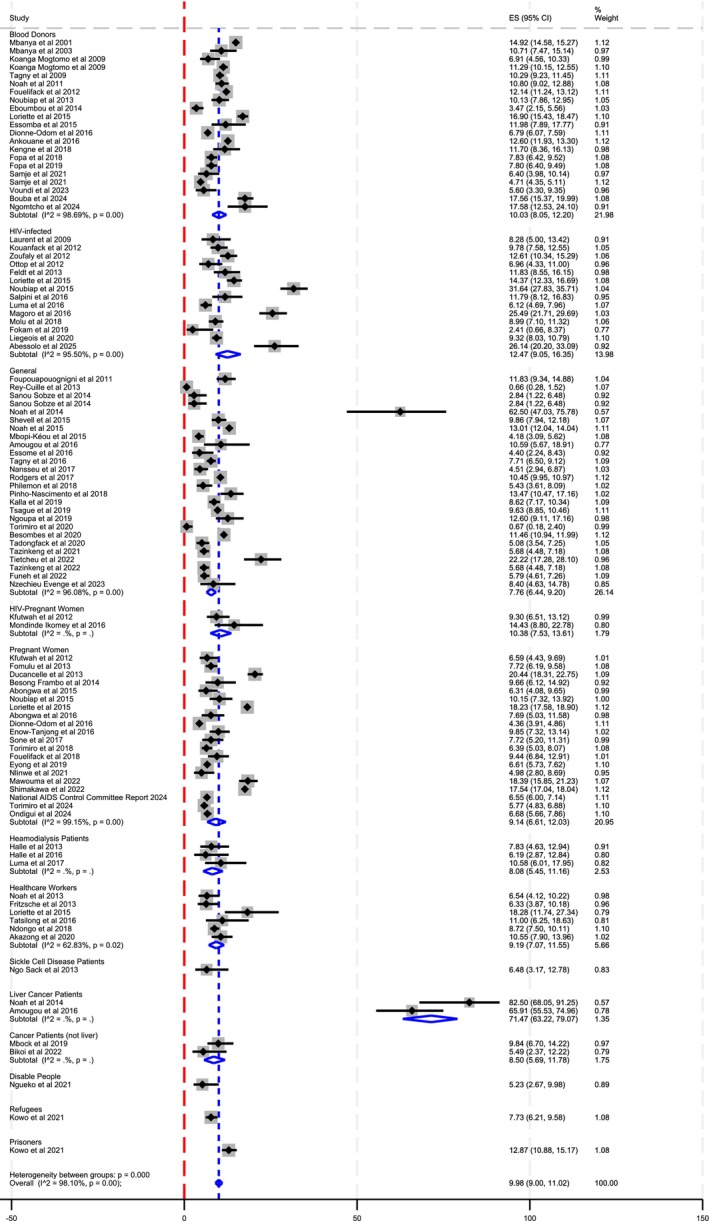
Forest plot for crude HBV prevalence stratified by population groups using all included studies.

In the pooled sample of 212,167 individuals across all included studies, the pooled overall prevalence of HBV was 9.98% (95% CI 9.00–11.02), with high heterogeneity (*I* ^*2*^ *= 98.10%*) (see Figure 2).

### 3.5 Sensitivity Analyses

In sensitivity analyses, our results suggest that the prevalence of HBV including only high‐quality studies was 9.73% (95% CI 7.69–11.98). For analysis, including only studies which used the ELISA screening method, the prevalence of HBV was 11.74% (95% CI 9.82–13.80). The analysis based on only population‐based studies indicated a prevalence of HBV of 9.08% (95% CI 7.65–10.62) (see Table S8).

### 3.6 Subgroup Analyses and Sources of Heterogeneity

Table 2 displays the prevalence of HBV infection in each subgroup. In a pooled sample of 198,267 individuals at lower‐risk of HBV infection, the pooled overall prevalence was 8.94% (95% CI 7.84–10.11) (*I* ^*2*^ *= 98.57%*) (see Figure S1). In a pooled sample of 13,900 individuals at higher‐risk of HBV infection, the pooled overall prevalence was 12.70% (95% CI 10.28–15.32) (*I* ^*2*^ *= 94.59%*) (see Figure S2).

**TABLE 2:** HBV prevalence subgroup analyses using random effects model by population.

Group	Studies	Cases	Participants	Prevalence of HBV (95% CI)	*I* ^*2*^, %	*p*‐*difference*
Sex						
Male	19	3936	40,787	10.44 (8.38–12.69)	97.66	0.035
Female	32	8707	68,312	8.85 (6.88–11.03)	98.69	
Age group						
≤ 15 years old	4	1630	15,296	2.81 (0.00–11.60)	98.90	0.137
> 15 years old	44	8445	76,813	9.14 (7.62–10.79)	98.00	
Settings						
Rural	23	9529	63,267	12.05 (10.14–14.10)	97.79	0.058
Urban	42	10,124	78,269	10.15 (8.81–11.58)	96.16	
Time period						
≤ 2005	7	6404	44,183	9.82 (7.41–12.54)	93.16	0.087
2006–2016	60	15,920	128,972	10.65 (9.36–12.02)	98.07	
≥ 2017	28	2535	36,462	8.40 (6.97–9.95)	95.21	
Population risk groups						
Lower risk groups	67	23,520	198,267	8.94 (7.84–10.11)	98.57	0.004
Higher risk groups	33	1639	13,900	12.70 (10.28–15.32)	98.10	
Regions						
Adamawa	2	104	626	16.40 (13.59–19.42)		< 0.001
Centre	36	9328	73,644	9.93 (8.32–11.67)	97.23	
East	1	75	970	7.73 (6.21–9.58)		
Far‐North	8	7224	41,026	16.86 (15.83–17.92)	74.30	
Littoral	8	554	5352	8.73 (6.45–11.30)	86.25	
North	1	259	1267	20.44 (18.31–22.75)		
North‐West	9	869	15,722	7.62 (5.54–9.99)	91.42	
West	6	597	7500	4.81 (2.59–7.65)	93.32	
South	2	1477	13,871	10.50 (9.99–11.02)		
South‐West	12	572	7959	8.01 (6.63–9.51)	77.06	
Study design						
Case‐control	4	125	253	54.35 (19.81–86.73)	96.95	< 0.001
Cross‐sectional	91	23,045	196,746	8.90 (7.92–9.92)	98.12	
Retrospective cross‐sectional	5	1989	15,168	15.49 (11.83–19.55)	96.55	

Sex, population risk groups, geographical area (regions) and study design were the main sources of heterogeneity. Our results suggest that the prevalence of HBV is higher in males (9.59%) compared to females (8.67%), and it is higher in the North region (20.44%) and lowest in the West region of Cameroon (4.81%). We also notice that the prevalence is higher in case‐control studies (24.40%) than cross‐sectional studies (8.54%) and among populations at higher‐risk of HBV infection. There is weak evidence of a difference in prevalence across age groups, settings or period of time that the study was undertaken. Similar trends were observed among the higher‐risk groups and the lower‐risk groups; however, the magnitude of differences varied. For example, although prevalence is higher among males than females in both lower‐risk (Female: 8.67% CI 6.30–11.38; Male: 9.59% CI 7.59–11.79) and higher‐risk groups (Female: 8.84% CI 7.50–10.28; Male: 16.61% CI 14.70–18.61), the difference in prevalence is larger, and the evidence of this difference is stronger among high‐risk groups. Full results of subgroup analyses for populations known to be at lower‐risk and at higher‐risk of HBV infection are given separately in supplementary tables (see Tables S6 and S7).

## 4 Discussion

### 4.1 Main Findings

A total of 92 papers describing 100 studies were included in this systematic review and meta‐analysis with an overall pooled chronic HBV prevalence of 9.98% (95% CI 9.00–11.02). There was high heterogeneity across studies. In subgroup analyses, the findings suggested that the prevalence of HBV infection was higher among higher‐risk groups 12.70% (95% CI 10.28–15.32) than lower‐risk groups 8.94% (95% CI 7.84–10.11). Prevalence also differed by sex in Cameroon with males (9.59%) having higher HBV prevalence than females (8.67%). The reasons for this variation are not well established but may be due to a combination of hormonal and biological factors [112, 113]. Also, findings suggested that there is weak evidence of a decrease in HBV prevalence between studies published in 2006–2016 and studies published later (post‐2017). The prevalence is also higher in the North region, in studies carried out in prison and case‐control studies. Our results also suggested a large variability in HBV prevalence across geographical areas (regions). The higher prevalence in some regions might be driven by being more rural. This would be consistent with several studies finding higher prevalence in rural compared to urban areas [24, 45, 71, 87]. Geographical regions represent broad administrative areas used to compare differences across locations within Cameroon, while the urban–rural division classifies areas by settlement type, infrastructure and level of development. The observed regional variation in HBV prevalence may partly reflect these urban–rural disparities, as regions with higher prevalence tend to be more rural, where limited healthcare access and services can contribute to increased infection rates. Future research would be valuable to further investigate this trend and whether it applies in Cameroon.

Sex, higher‐risk population groups, regions in Cameroon and study design were the main sources of heterogeneity. After excluding poor and moderate studies in sensitivity analyses, the HBV prevalence was 9.73% (95% CI 7.69–11.98), which was similar to the overall prevalence in the main analysis.

The overall pooled prevalence in this study over the period of 2000–2025 (9.98%) is lower than that reported in the previous meta‐analysis covering the period 2000–2016 (11.2%) [5], although a sensitivity analysis limited to studies using ELISA yielded a higher prevalence of 11.74%. This suggests that variations in screening methods may influence the estimates. Since ELISA is considered to be more accurate, the lower overall prevalence observed by including all eligible studies may partly result from the inclusion of studies using less sensitive diagnostic tools. However, while it is difficult to conclude with certainty that there has been a true decline in HBV prevalence, as the observed difference may be driven by methodological factors, the prevalence when including only studies from 2017 to 2025 (i.e., post‐Bigna et al.) that use ELISA is 9.22% (95% CI 7.19–11.49). Since this is lower than the comparable figure of 11.74% from ELISA‐based studies over the whole period, it provides further support that there has been a decline in prevalence across more recently published studies compared to previous ones. Following classification by the MoH [4], we have regions with lower prevalence (< 8%: North‐West and West), intermediate prevalence (8%–15%: Centre, Littoral, South and South‐West) and high prevalence (> 15%: Adamawa, Far‐North and North) [4]. From the results of these categories, the three regions in the northern part (Adamawa, Far‐North and North) alone reported the highest prevalences in the country. Therefore, specific plans for the three northern regions with the highest prevalences are urgently required to better understand the risk factors associated with the spread of HBV infection in these regions. Further investigation is needed to develop an understanding of the drivers of high prevalence across the country and the variation across the regions to inform policy action.

### 4.2 Implications of the Study

The overall prevalence estimate of 9.98% (95% CI 9.00–11.02) is slightly lower, by 1.22 percentage points, than that found in a previous systematic review and meta‐analysis published in 2017, which reported an overall prevalence of 11.20% (95% CI 9.7–12.8) [5]. Our subgroup analysis on study time period suggested weak evidence of a decrease in HBV prevalence in more recently published studies. In our study, we estimated a 2.25 percentage point reduction from 2006 to 2016 (10.65%) [5] to 2017–2025 (8.40%), although we note a slight overlap in the confidence intervals. We speculate that this observed decrease could be a result of efforts over the years from the government in Cameroon to decrease prevalence [4, 6]. For example, systematic screening measures are recommended for all first‐time blood donors and all pregnant women attending antenatal care visits should be screened, although this remains a paid‐for service [4, 6].

The pooled prevalence of 8.94% (95% CI 7.84–10.11) found among lower‐risk groups in Cameroon is similar to the prevalence of 8.83% (95% CI 8.82–8.83) found in the African region from a global review of HBV prevalence published in 2015 [114]. With a total population of 29 million inhabitants in Cameroon in 2025 [115] and from the prevalence we found in this study, it is estimated that 2.9 million Cameroonians carry HBV. Despite progress in the diagnosis of HBV infections since 2017, which has increased the proportion of HBV infections that are diagnosed, many infections still do not get diagnosed and so treatment cannot be sought. This is a problem across Cameroon, but it is likely to be more acute in rural areas as the majority of services are concentrated in urban areas [116, 117]. Consequently, there is still an urgent need to increase access to screening tests, expanding beyond urban areas, vaccination efforts and treatment across the country to eliminate HBV by 2030. A National Control Programme focusing on viral hepatitis would also be needed in Cameroon to coordinate surveillance activities as well as prevention measures and treatment to fight against HBV infection. The findings obtained from this study may be used to increase awareness of HBV in communities by carrying out health campaigns, including free screening initiatives in rural and urban areas, as well as providing policymakers with some evidence for them to make informed decisions as they refine their roadmap and strategies against HBV. All these efforts can impact population health and prevent the transmission of the disease. In addition, the findings of this study could point to the need for additional interventions from government, for example, the treatment of HBV being subsidized by the MoH and making medicines available in approved treatment centres in all regions [118]. Cameroon now has 20 treatment centres approved by the MoH where medicines against viral hepatitis are distributed. Half of these centres are based in two major cities: Yaounde and Douala; however, from this review, we found some weak evidence that the prevalence of HBV may be higher in rural areas than urban areas. Despite the government's effort, it is estimated in 2017 that only around 8% of people with HBV are receiving treatment, making disease control and elimination arduous since there is no national control programme on viral hepatitis to coordinate surveillance and management activities [6, 118]. The findings of this study indicate that prevalence remains high and so additional interventions may be required.

Existing HBV interventions in Cameroon include targeted birth dose vaccination, administered only to newborns of HBsAg‐positive mothers, followed by free pentavalent vaccine doses given at 6, 10 and 14 weeks. Additional measures include antiviral treatment of HBV at reduced out‐of‐pocket costs for patients living with HBV to fight the disease. In addition, antenatal screening helps identify infected pregnant women to guide prevention. Key recommendations to expand interventions include universal birth dose vaccination for all newborns, free screening of adults in Cameroon, including women during antenatal care, vaccinating uninfected adults, and improving service accessibility and affordability nationwide. These strategies aim to increase coverage, reduce transmission and enhance overall cost‐effectiveness. Targeting high‐risk population groups through interventions is essential because they have higher HBV prevalence and greater exposure to infection. Intervening in these groups may improve efficiency, reduce transmission, prevent severe outcomes and address inequalities.

### 4.3 Strengths and Limitations

As far as we know, this is the second systematic review and meta‐analysis in Cameroon focusing on HBV infection. This review updates a previous one published in 2017 [5], and it includes new studies published since then, presenting a more up‐to‐date estimate of HBV prevalence in Cameroon. In addition to the overall prevalence, this review gives estimates of prevalence in a range of different population groups (general population, blood donors, pregnant women, HIV‐infected, HIV‐pregnant women, haemodialysis patients, healthcare workers, sickle cell disease patients, people with disabilities, prisoners, refugees, liver cancer patients and patients with any other type of cancer). These up‐to‐date and disaggregated estimates of prevalence are essential to inform effective targeted policy and intervention to tackle the high prevalence and variation in prevalence across the population.

There are a number of limitations to this study. We must highlight that 36% of the included studies in the meta‐analysis were carried out only in one out of the 10 regions in Cameroon (Centre), where around 12% of the total population live. This may affect the generalizability of the results since that region contributes disproportionately to the pooled prevalence. In terms of methodological quality, slightly more than a third of the 100 included studies from the meta‐analysis had poor quality assessment. However, in the sensitivity analyses, we found that including only high‐quality studies resulted in a similar prevalence as the main analysis. Also, although comprehensive searches were undertaken including some grey literature from Cameroonian sources, it is possible that other grey literature estimating the HBV prevalence in Cameroon may have been missed. In addition, our findings may need to be interpreted with caution because we have more studies among females compared to males and fewer studies in some regions of the country compared to others.

## 5 Conclusion

HBV prevalence remains high among the general population and across other population groups in Cameroon. Particular attention is required for males and regions with the highest prevalences (North, Far‐North and Adamawa). The data and the results we presented in this systematic review and meta‐analysis are important to frame an up‐to‐date picture of the variability of prevalences across population groups and regions, which are necessary for planning HBV interventions. The findings suggest the urgent need for interventions to reduce prevalence and the spread of disease if the MoH in Cameroon is to achieve WHO targets to eliminate HBV as a public health problem by 2030.

## Author Contributions

Iya Maliki conceptualized the study and developed the protocol with support from Bryony Dawkins, Edward J.D. Webb and Aaron G. Lim. Iya Maliki developed the search strategy with support from Bryony Dawkins, Edward J.D. Webb and Aaron G. Lim. Iya Maliki conducted the database searching, and double‐screened titles/abstracts and full texts with Rose Armelle Ada. Iya Maliki extracted the data with an Excel sheet, ran all analyses and interpreted the results with support from Bryony Dawkins, Edward J.D. Webb and Aaron G. Lim. Iya Maliki drafted the original article with support from Bryony Dawkins, Edward J.D. Webb and Aaron G. Lim. All authors revised, commented and edited the manuscript, and the final version was approved by all.

## Funding

This project is funded by the Commonwealth Scholarship Commission (CSC) in the UK through the Foreign, Commonwealth and Development Office (FCDO), UK government. In this publication, all views expressed are those of the authors and not those of the CSC or the FCDO.

## Ethics Statement

This study did not involve human research participants; therefore, ethics approval and participant consent were not required.

## Conflicts of Interest

The authors declare no conflicts of interest.

## Supporting information


**Data S1:** Supporting Information.

**Table S1:** Databases search strategies.
**Table S2:** Eligibility criteria.
**Table S3:** Joanna Briggs Institute critical appraisal criteria.
**Table S4:** Age categorization in years.
**Figure S1:** Forest plot for HBV prevalence at low risk of HBV infection.
**Figure S2:** Forest plot for HBV prevalence at high risk of HBV infection.
**Table S6:** HBV prevalence subgroup analyses among lower risk groups using random effects model.
**Table S7:** HBV prevalence subgroup analyses among higher risk group using random effects model.
**Table S8:** HBV prevalence: Sensitivity analyses for quality assessment among high quality.
**Figure S3:** Number of yes, no, unclear and not applicable to each Joanna Briggs Institute quality assessment (N = 100).
**Table S5:** List of 100 studies included from 92 papers in this systematic review and meta‐analysis.

## Data Availability

The data that supports the findings of this study are available in the supporting information of this article.
