# Supplementary material for: Prevalence of Hepatitis B Virus Infection in Cameroon: A Systematic Review and Meta‐Analysis
Source: J Viral Hepat. 2026 Aug 5;33(9):e70219. doi: 10.1111/jvh.70219 (PMC13439727; doi:10.1111/jvh.70219)
Supplement: Supplementary file 2 [file JVH-33-0-s002.docx]

**Prevalence of Hepatitis B Virus Infection in Cameroon: A systematic review and meta-analysis**

Iya Maliki^1*^, Edward JD Webb^1^, Rose Armelle Ada^2^, Bryony Dawkins^1†^, Aaron G. Lim^3†^

**Authors affiliations**

^1^Academic Unit of Health Economics, Leeds Institute of Health Sciences, University of Leeds, UK.

^2^Department for the Control of Disease, Epidemics and Pandemics, Ministry of Public Health, Cameroon.

^3^Population Health Sciences, Bristol Medical School, University of Bristol, UK.

^†^ Joint senior author

**Correspondence***

Iya Maliki, Academic Unit of Health Economics, Leeds Institute of Health Sciences, University of Leeds, LS2 9JT, UK

Email: [umim@leeds.ac.uk](mailto:umim@leeds.ac.uk)

1. **Methods**
   1.1. **Search strategy**

“Hepatitis B virus” and “Cameroon” are the main two concepts of the search strategy. We developed the search strategy by looking at published systematic reviews and meta-analysis on the topic, databases, and literature. The final search strategy was reviewed and approved by an information specialist. All searches were performed without language restriction from the 1^st^ of January 2000 as Bigna et al in 2017 we are updating to 10^th^ February 2025, the date our searches were conducted. We used key words, Boolean operators, and Medical Subject Headings (see **Supplementary Table S1)**.

**Supplementary Table S1:** Databases search strategies

**Ovid MEDLINE(R) ALL <1946 to February 07, 2025>**

1	exp Hepatitis B/	67307

2	Hepatitis B virus/	34113

3	(infect* adj2 HBV).tw.	23681

4	(hepatitis b or jaundice or Australia antigen or hepatitis d*).tw.	135736

5	(HBV or VHB or CHB or HBsAg or HBS-ag or HBcAb or HBsAb or HBeAg).tw.	66992

6	Jaundice/	13819

7	exp Hepatitis B Antigens/	34570

8	1 or 2 or 3 or 4 or 5 or 6 or 7	168570

9	Cameroon/	6993

10	cameroon*.ti,ab,in.	16476

11	(douala or Ngaoundere or Yaounde or Maroua).ti,ab,in.	10857

12	9 or 10 or 11	17990

13	8 and 12	281

14	limit 13 to yr="2000 -Current"	255

**Global Health <1910 to 2025 Week 06>**

1	exp Hepatitis B virus/	45110

2	(infect* adj2 HBV).tw.	13252

3	(hepatitis b or jaundice or Australia antigen or hepatitis d*).tw.	61236

4	(HBV or VHB or CHB or HBsAg or HBS-ag or HBcAb or HBsAb or HBeAg).tw.	33022

5	Jaundice/	7674

6	1 or 2 or 3 or 4 or 5	62521

7	Cameroon/	9600

8	cameroon*.ti,ab,in.	12354

9	(douala or Ngaoundere or Yaounde or Maroua).ti,ab,in.	6597

10	7 or 8 or 9	13399

11	6 and 10	231

12	limit 11 to yr="2000 -Current"	199

**Embase <1996 to 2025 Week 06>**

1	exp Hepatitis B/	113740

2	exp Hepatitis B virus/	55944

3	(infect* adj2 HBV).tw.	33509

4	(hepatitis b or jaundice or Australia antigen or hepatitis d*).tw.	157729

5	(HBV or VHB or CHB or HBsAg or HBS-ag or HBcAb or HBsAb or HBeAg).tw.	94316

6	Jaundice/	42202

7	exp Hepatitis B Antigens/	51996

8	1 or 2 or 3 or 4 or 5 or 6 or 7	234470

9	Cameroon/	8602

10	cameroon*.ti,ab,in.	18866

11	(douala or Ngaoundere or Yaounde or Maroua).ti,ab,in.	13121

12	9 or 10 or 11	19734

13	8 and 12	405

14	limit 13 to yr="2000 -Current"	402

**Cochrane**

Search Name:	SRMACMRFinal

Date Run:	10/02/2025 19:07:21

Comment:

ID	Search	Hits

#1	MeSH descriptor: [Hepatitis B Antigens] explode all trees	1424

#2	MeSH descriptor: [Hepatitis B] explode all trees	3563

#3	MeSH descriptor: [Hepatitis B virus] explode all trees	1118

#4	MeSH descriptor: [Jaundice] explode all trees	260

#5	(infect* near/2 HBV):ti,ab,kw	1355

#6	(hepatitis b or jaundice or Australia antigen or hepatitis d*):ti,ab,kw	24866

#7	(HBV or VHB or CHB or HBsAg or HBS-ag or HBcAb or HBsAb or HBeAg):ti,ab,kw	6888

#8	#1 or #2 or #3 or #4 or #5 or #6 or #7	25908

#9	(Cameroon*):ti,ab,kw	437

#10	MeSH descriptor: [Cameroon] explode all trees	164

#11	(Douala or Ngaoundere or Yaounde or Maroua):ti,ab,kw	115

#12	#9 or #10 or #11	452

#13	#8 and #12	8

**# Web of Science Search Strategy (v0.1)**

\# Database: Web of Science Core Collection

\# Entitlements:

- WOS.SCI: 1900 to 2025

- WOS.AHCI: 1975 to 2025

- WOS.ESCI: 2015 to 2025

- WOS.ISTP: 1990 to 2025

- WOS.SSCI: 1900 to 2025

- WOS.ISSHP: 1990 to 2025

\# Searches:

1: TS=((infect* near/2 HBV))	Date Run: Mon Feb 10 2025 15:12:51 GMT+0000 (Greenwich Mean Time)	Results: 24752

2: TS=((hepatitis b or jaundice or Australia antigen or hepatitis d or hepatitis delta))	Date Run: Mon Feb 10 2025 15:13:13 GMT+0000 (Greenwich Mean Time)	Results: 183926

3: TS=(((HBV or VHB or CHB or HBsAg or HBS-ag or HBcAb or HBsAb or HBeAg)))	Date Run: Mon Feb 10 2025 15:13:32 GMT+0000 (Greenwich Mean Time)	Results: 75077

4: #3 OR #2 OR #1	Date Run: Mon Feb 10 2025 15:13:45 GMT+0000 (Greenwich Mean Time)	Results: 202032

5: ALL=( cameroon* or douala or Ngaoundere or Yaounde or Maroua)	Date Run: Mon Feb 10 2025 15:14:25 GMT+0000 (Greenwich Mean Time)	Results: 42416

6: #5 AND #4	Date Run: Mon Feb 10 2025 15:16:54 GMT+0000 (Greenwich Mean Time)	Results: 376

7: #5 AND #4	Timespan: 2000-01-01 to 2025-02-10	Date Run: Mon Feb 10 2025 15:17:42 GMT+0000 (Greenwich Mean Time)	Results: 337

**African Index Medicus**

(((hepatitis b OR hepatitis d OR jaundice OR hbv OR vhb OR chb OR hbsag OR hbs-ag OR hbcab OR hbsab OR hbeag))) AND ((cameroon* OR douala OR ngaoundere OR yaounde OR maroua)) AND ( collection_gim:("AIM")) AND (year_cluster:[2000 TO 2025])

exp stands for explodes. It was used to capture all narrower terms associated with the broader concept in Ovid Medline, Global Health and Embase;

tw was used to find word or text in the title and abstract field;

topic was used to find word or text in the title, abstract, and keywords field in Web of Science;

* was used to include various word endings and spellings;

AND was used to find results that contain both or all;

OR was used to find results that contain either.

1.2. **Eligibilty Criteria**

All studies on the prevalence of Hepatitis B virus in Cameroon in any language were included (see **Supplementary Table S2).**

**Supplementary Table S2:** Eligibility criteria

| **N** | Inclusion criteria | Exclusion criteria |
| --- | --- | --- |
| **1** | Observational studies (cross-sectional, case-control, or cohort studies) among Cameroonians (males and females), | Studies conducted among Cameroonians outside the country, |
| **2** | Community-based surveys, | Animal studies, |
|  | Chronic hepatitis B virus infection defined by the presence of hepatitis B surface antigen (HBsAg)s, | Study designs in clinical trials, author reply, case reports, qualitative studies intervention studies, systematic reviews, editorials, commentaries, letters to editor and conference abstract and studies we could not find the full text, |
| **3** | - | Studies reporting prevalence without providing sufficient information to extract the numerator and denominator |

1.3. **Critical Appraisal**

The Joanna Briggs Institute (JBI) critical appraisal tool for prevalence studies with nine questions to evaluate the validity (internal and external) of a study was used for quality assessment of each of the study. Each question has four possibles answers (Yes, No, Unclear, and Not applicable). Following discussion with the supervisory team, we established that zero to four yeses, five to six yeses and seven to nine yeses would be considered as poor, moderate and high quality respectively after summing the number of yeses. The first four questions are important to assess external validity on setting, description of study subjects, sample size, study subject selection method and sampling frame appropriateness. The remaining five questions are used to assess the internal validity of the study which enables us to assess the adequacy of the response rate, the use of valid methods to identify HBV, the use of proper statistical analysis and data analysis for all subgroups (see **Supplementary Table S3)**.

**Supplementary Table S3:** Joanna Briggs Institute critical appraisal criteria

| **Questions** | **Yes** | **No** | **Unclear** | **Not Applicable** |
| --- | --- | --- | --- | --- |
| 1. Was the sample frame appropriate to address the target population? | □ | □ | □ | □ |
| 2. Were study participants sampled in an appropriate way? | □ | □ | □ | □ |
| 3. Was the sample size adequate? | □ | □ | □ | □ |
| 4. Were the study subjects and the setting described in detail? | □ | □ | □ | □ |
| 5. Was the data analysis conducted with sufficient coverage of the identified sample? | □ | □ | □ | □ |
| 6. Were valid methods used for the identification of the condition? | □ | □ | □ | □ |
| 7. Was the condition measured in a standard, reliable way for all participants? | □ | □ | □ | □ |
| 8. Was there appropriate statistical analysis? | □ | □ | □ | □ |
| 9. Was the response rate adequate, and if not, was the low response rate managed appropriately? | □ | □ | □ | □ |

1.4. **Age classification**

Age grouping across all the included studies was not possible. The common age group reported across the studies was 15 years old and below, and above 15 years old. There was also an age grouping that crossed the above age cutting value. For studies having the upper age range, the lower age range, and the mean age, we categorized according to which this mean lays (see **Supplementary Table S4)**.

**Supplementary Table S4**: Age categorization in years

| Ages grouped under ≤ 15 | Ages grouped 15 and above |
| --- | --- |
| ≤ 15 | >15 |
| ≤ 15,  0 to 6,  0 to 15,  0 to 5 | >15, >15, >15, >15, >15, >15, ≥18, ≥21, ≥20, 18 to 60, 18 to 58, 16 to 69, 16 to 43, 21 to 57, 18 to 62, 5 to 47, 20 to 60, 18 to 60, 15 to 42, 8 to 77, 11 to 82, 13 to 44, 15 to 40, 18 to 94, 18 to 23, 19 to 56, 17 to 44, 8 to 93, 18 to 48, 16 to 47, 16 to 59, 20 to 43, 1 to 92, 3 to 88, 18 to 43, 4 to 93, 18 to 52, 18 to 65, 19 to 91, 20 to 85, 14 to 47, 16 to 65, 2 to 87, 14 to 74, 18 to 57, 15 to 49, 15 to 48, 19 to 78, 21 to 80, 18 to 60, 15 to 49, 18 to 60, 15 to 45, 10 to 40 |

2. **Results**

The Forest Plot of the prevalence of HBV infection in populations known to be at low risk of HBV infection (general population, blood donors, and pregnant women) is showed below. (see **Supplementary Figure S1)**. The pooled prevalence is 8.94% (95% CI 7.84 to 10.11) with studies among blood donors had a prevalence of 10.03 (95% CI 8.06, 112.20) and studies among general population had a prevalence of 7.76 (95% CI 6.44, 9.20) which represents the highest and lowest prevalence respectively.

**Supplementary Figure S1:** Forest plot for HBV prevalence at low risk of HBV infection

The Forest Plot of the prevalence of HBV infection in populations known to be at high risk of HBV infection (healthcare workers, HIV-infected, HIV-pregnant women, heamodialysis patients, sickel cell disease patients, liver cancer patients, non-liver cancer patient, prisoners, refugees, and disabled people) is showed below (see **Supplementary Figure S2)**. The pooled prevalence is 12.70 (95% CI 10.28, 15.32) with studies among patients with liver cancers had a prevalence of 71.44 (95% CI 63.22, 79.07) and studies among disabled people had a prevalence of 5.23 (95% CI 2.67, 9.98) which represents the highest and lowest prevalence respectively.

**Supplementary Figure S2:** Forest plot for HBV prevalence at high risk of HBV infection

**Supplementary Table S6:** HBV prevalence subgroup analyses among lower risk groups using random effects model

| **Group** | **Studies** | **Cases** | **Participants** | **Prevalence of HBV, (95%CI)** | **I^2^, %** | **p-*difference*** |
| --- | --- | --- | --- | --- | --- | --- |
| **Sex** |  |  |  |  |  |  |
| Male | 17 | 3699 | 39392 | 9.59 (7.59, 11.79) | 97.60 | 0.585 |
| Female | 22 | 8308 | 63777 | 8.67 (6.30, 11.38) | 99.08 |  |
| **Age group** |  |  |  |  |  |  |
| ≤15 years old | 35 | 8034 | 72173 | 9.25 (7.50, 11.15) | 98.39 | 0.201 |
| > 15 years old | 3 | 1628 | 15213 | 2.95 (0.00, 13.74) | . |  |
| **Settings** |  |  |  |  |  |  |
| Rural | 17 | 9097 | 59631 | 11.58 (9.40, 13.20) | 98.23 | 0.058 |
| Urban | 27 | 9701 | 74811 | 8.96 (7.56, 10.46) | 96.77 |  |
| **Time period** |  |  |  |  |  |  |
| ≤ 2005 | 5 | 6362 | 43713 | 10.18 (7.40, 13.33) | 94.61 | 0.343 |
| 2006 to 2016 | 39 | 14778 | 119282 | 9.24 (7.82, 10.77) | 98.53 |  |
| ≥2017 | 20 | 2212 | 33407 | 7.99 (6.46, 9.67) | 95.74 |  |
| **Regions** |  |  |  |  |  |  |
| Adamawa | 2 | 104 | 626 | 16.40 (13.59, 19.42) | . | <0.001 |
| Centre | 23 | 8986 | 70684 | 8.39 (6.71, 10.23) | 97.79 |  |
| Far-North | 6 | 7064 | 39938 | 17.26 (16.26, 18.30) | 74.24 |  |
| Littoral | 5 | 414 | 4149 | 8.40 (5.35, 12.05) | 90.23 |  |
| North | 1 | 259 | 1267 | 20.44 (18.31, 22.75) | . |  |
| North-West | 6 | 707 | 14355 | 5.51 (4.57, 6.53) | 47.33 |  |
| West | 6 | 597 | 7500 | 4.81 (2.59, 7.65) | 93.32 |  |
| South | 1 | 1431 | 13695 | 10.45 (9.95, 10.97) | . |  |
| South-West | 8 | 476 | 6562 | 8.34 (6.57, 10.30) | 82.72 |  |
| **Study design** |  |  |  |  |  |  |
| Case control | 2 | 34 | 125 | 24.40 (17.14, 32.43) | . | <0.001 |
| Cross sectional | 63 | 21785 | 184474 | 8.54 (7.40, 9.75) | 98.64 |  |
| Retrospective cross sectional | 2 | 1701 | 13668 | 12.44 (11.90, 13.00) | . |  |

**Supplementary Table S7:** HBV prevalence subgroup analyses among higher risk group using random effects model

| **Group** | **Studies** | **Cases** | **Participants** | **Prevalence of HBV, (95%CI)** | **I^2^, %** | **p-*difference*** |
| --- | --- | --- | --- | --- | --- | --- |
| **Sex** |  |  |  |  |  |  |
| Male | 2 | 237 | 1395 | 16.61 (14.70, 18.61) | . | <0.001 |
| Female | 10 | 399 | 4535 | 8.84 (7.50, 10.28) | 53.11 |  |
| **Age group** |  |  |  |  |  |  |
| ≤15 years old | 1 | 2 | 83 | 2.41 (0.66, 8.37) | . | 0.023 |
| > 15 years old | 9 | 411 | 4640 | 8.70 (7.00, 10.56) | 72.46 |  |
| **Settings** |  |  |  |  |  |  |
| Rural | 6 | 432 | 3636 | 13.58 (9.75, 17.91) | 91.43 | 0.862 |
| Urban | 15 | 423 | 3458 | 14.01 (9.12, 19.73) | 94.61 |  |
| **Time period** |  |  |  |  |  |  |
| ≤ 2005 | 2 | 42 | 470 | 8.92 (6.48, 11.69) | . | 0.037 |
| 2006 to 2016 | 21 | 1142 | 9690 | 14.33 (10.93, 18.09) | 95.62 |  |
| ≥2017 | 8 | 323 | 3055 | 9.55 (6.39, 13.25) | 95.62 |  |
| **Regions** |  |  |  |  |  |  |
| Centre | 13 | 342 | 2960 | 14.57 (8.88, 21.34) | 95.36 | <0.001 |
| Far-North | 2 | 160 | 1088 | 14.55 (12.49, 16.73) | . |  |
| East | 1 | 75 | 970 | 7.73 (6.21, 9.58) | . |  |
| Littoral | 3 | 140 | 1203 | 9.43 (5.63, 14.06) | . |  |
| North-West | 3 | 162 | 1367 | 11.82 (10.16, 13.60) | . |  |
| South | 1 | 46 | 176 | 26.14 (20.20, 33.09) | . |  |
| South-West | 4 | 96 | 1397 | 7.38 (5.06, 10.08) | 58.81 |  |
| **Study design** |  |  |  |  |  |  |
| Case control | 2 | 91 | 128 | 71.47 (63.22, 79.07) | . | <0.001 |
| Cross sectional | 28 | 1260 | 12272 | 9.85 (8.33, 11.49) | 86.69 |  |
| Retrospective cross sectional | 3 | 288 | 1500 | 17.97 (7.36, 31.91) | . |  |

**Supplementary Table S8:** HBV prevalence: sensitivity analyses for quality assessment among high quality

| **Group** | **Studies** | **Cases** | **Participants** | **Prevalence of HBV, (95%CI)** | **I^2^, %** | **p-*difference*** |
| --- | --- | --- | --- | --- | --- | --- |
| **Quality of assessment** |  |  |  |  |  |  |
| High quality studies | 19 | 11390 | 86845 | 9.73 (7.69, 11.98) | 98.97 | <0.001 |
| **Screening method** |  |  |  |  |  |  |
| ELISA | 25 | 8570 | 64484 | 11.74 (9.82, 13.80) | 97.83 | <0.001 |
| **Sites of study** |  |  |  |  |  |  |
| Population-based | 10 | 4041 | 37673 | 9.08 (7.65, 10.62) | 94.61 | <0.001 |

**Responses to the nine Joanna Briggs Institute quality assessment questions**

|  | Yes | No | Unclear | Not Applicable |
| --- | --- | --- | --- | --- |
| 1. Sample frame appropriate | 62 | 21 | 17 | 0 |
| 2. Sampled in an appropriate way | 48 | 15 | 37 | 0 |
| 3. Sample size adequate? | 38 | 22 | 40 | 0 |
| 4. Subjects and the setting described in detail | 45 | 31 | 24 | 0 |
| 5. Data analysis conducted with sufficient | 42 | 17 | 41 | 0 |
| 6. Valid methods used for the identification of the condition | 51 | 28 | 18 | 3 |
| 7. Condition measured in a standard and reliable way | 39 | 17 | 42 | 2 |
| 8. Appropriate statistical analysis performed | 40 | 23 | 31 | 6 |
| 9. Response rate adequate | 38 | 19 | 39 | 4 |

**Supplementary Figure S3:** Number of yes, no, unclear and not applicable to each Joanna Briggs Institute quality assessment **(N=100)**.

**Supplementary Table S5:** List of 100 studies included from 92 papers in this systematic review and meta-analysis

| **N** | **Author** | **Year** | **Start** | **End** | **Region** | **Setting** | **Site** | **Population** | **Design** | **Cases** | **Sample Size** | **Prevalence, %** | **Mean** | **Age range, years** | **Age group** | **Sex** | **%Male** | **Screening tool** | **Risk of Bias** |
| --- | --- | --- | --- | --- | --- | --- | --- | --- | --- | --- | --- | --- | --- | --- | --- | --- | --- | --- | --- |
| 1 | Mbanya et al | 2001 | 1994 | 1998 | Centre | Urban | Hospital-based | Blood Donors | CS | 5989 | 40134 | 14.9 | NR | NR | NR | M & F | 40.8 | NR | Moderate |
| 2 | Mbanya et al | 2003 | 2001 | 2001 | Centre | Urban | Hospital-based | Blood Donors | CS | 27 | 252 | 10.7 | NR | 18-60 | Beyond 15 | M & F | 78.2 | RDT | Moderate |
| 3 | Koanga Mogtomo et al | 2009 | 1995 | 2003 | Littoral | Urban | Hospital-based | Blood Donors | CS | 21 | 304 | 6.9 | NR | NR | NR | M & F | NR | NR | Poor |
| 4 | Koanga Mogtomo et al | 2009 | 1995 | 2003 | Littoral | Urban | Hospital-based | Blood Donors | CS | 302 | 2674 | 11.3 | NR | NR | NR | M & F | NR | NR | Poor |
| 5 | Tagny et al | 2009 | 2006 | 2007 | Centre | Urban | Hospital-based | Blood Donors | CS | 297 | 2887 | 10.3 | NR | 18-NR | Beyond 15 | M & F | 72 | RDT | Moderate |
| 6 | Laurent et al | 2009 | 2001 | 2003 | Centre | Urban | Hospital-based | HIV-infected | CS | 14 | 169 | 8.28 | 35 | NR | NR | M & F | 33.13 | EIA | High |
| 7 | Foupouapouognigni et al | 2011 | 2005 | 2008 | Centre, East, South | Rural | Population-based | General | CS | 62 | 524 | 11.83 | NR | NR | NR | M & F | NR | EIA | Poor |
| 8 | Noah et al | 2011 | 2007 | 2008 | Centre | Urban | Hospital-based | Blood Donors | CS | 108 | 1000 | 10.8 | 36.5 | 18-58 | Beyond 15 | M & F | 83.9 | ELISA | Poor |
| 9 | Kouanfack et al | 2012 | 2006 | 2007 | Centre | Urban | Hospital-based | HIV-infected | CS | 54 | 552 | 9.8 | NR | NR | NR | M & F | 29 | EIA | Moderate |
| 10 | Zoufaly et al | 2012 | 2005 | 2010 | Northwest | Rural | Hospital-based | HIV-infected | RCS | 87 | 690 | 12.6 | 35 | 15-NR | Beyond 15 | M & F | 35.2 | RDT | Poor |
| 11 | Fouelifack et al | 2012 | 2008 | 2008 | Centre | Urban | Hospital-based | Blood Donors | RCS | 564 | 4644 | 12.1 | 28 | 16-69 | Beyond 15 | M & F | 93.37 | NR | Poor |
| 12 | Kfutwah et al | 2012 | 2000 | 2003 | Centre | Urban | Hospital-based | HIV-Pregnant Women | CS | 28 | 301 | 9.3 | 26.2 | NR | NR | F | 0 | ELISA | Poor |
| 13 | Kfutwah et al | 2012 | 2000 | 2003 | Centre | Urban | Hospital-based | Pregnant Women | CS | 23 | 349 | 6.6 | 26.2 | NR | NR | F | 0 | ELISA | Poor |
| 14 | Halle et al | 2013 | 2012 | 2012 | Littoral | Urban | Hospital-based | Heamodialysis Patients | CS | 13 | 166 | 7.8 | 49.2 | NR | NR | M & F | 63.9 | ELISA | Moderate |
| 15 | Fomulu et al | 2013 | 2011 | 2012 | Centre | Urban | Hospital-based | Pregnant Women | CS | 74 | 959 | 7.7 | 27.6 | 15-43 | Beyond 15 | F | 0 | EIA | Moderate |
| 16 | Noah et al | 2013 | 2011 | 2011 | Centre | Urban | Hospital-based | Healthcare Workers | CS | 17 | 260 | 6.5 | 40.69 | 21-57 | Beyond 15 | M & F | 30 | RDT | Moderate |
| 17 | Noubiap et al | 2013 | 2011 | 2012 | Littoral | Urban & Rural | Hospital-based | Blood Donors | CS | 55 | 543 | 10.1 | 32.5 | 18-62 | Beyond 15 | M & F | 81.9 | RDT | Poor |
| 18 | Feldt et al | 2013 | 2009 | 2010 | Northwest | Urban & Rural | Hospital-based | HIV-infected | RCS | 33 | 279 | 11.8 | 38.1 | NR | NR | M & F | 34 | RDT | High |
| 19 | Ottop et al | 2012 | NR | NR | Southwest | Urban & Rural | Hospital-based | HIV-infected | CS | 16 | 230 | 6.9 | NR | NR | NR | M & F | 18 | RDT | High |
| 20 | Rey-Cuille et al | 2013 | 2009 | 2010 | Centre | Urban | Hospital-based | General | CS | 5 | 763 | 0.6 | NR | 0-6 | Under 15 | M & F | 43 | EIA | High |
| 21 | Ducancelle et al | 2013 | 2009 | 2010 | North | Rural | Hospital-based | Pregnant Women | CS | 259 | 1267 | 20.3 | NR | NR | NR | F | 0 | RDT | Moderate |
| 22 | Ngo Sack et al | 2013 | 2008 | 2009 | Centre | Urban | Hospital-based | Sickle Cell Disease Patients | CS | 7 | 108 | 6.5 | 21.45 | 5-47 | Beyond 15 | M & F | 52.8 | RDT | Poor |
| 23 | Fritzsche et al | 2013 | 2011 | 2011 | Southwest | Urban & Rural | Hospital-based | Healthcare Workers | CS | 15 | 237 | 6.3 | 34 | 20-60 | Beyond 15 | M & F | 31 | ELISA | Moderate |
| 24 | Eboumbou et al | 2014 | 2012 | 2012 | Littoral | Urban | Hospital-based | Blood Donors | CS | 16 | 461 | 3.5 | 30.2 | 18-60 | Beyond 15 | M & F | 80 | RDT & ELISA | Moderate |
| 25 | Sanou Sobze et al | 2014 | 2013 | 2013 | West | Urban | Hospital-based | General | CS | 5 | 176 | 2.8 | 23.3 | NR | NR | M & F | NR | RDT | Moderate |
| 26 | Besong Frambo et al | 2014 | 2012 | 2012 | Southwest | Urban & Rural | Hospital-based | Pregnant Women | CS | 17 | 176 | 9.7 | 25.7 | 15-42 | Beyond 15 | F | 0 | RDT | Poor |
| 27 | Sanou Sobze et al | 2014 | 2013 | 2013 | West | Rural | Hospital-based | General | CS | 5 | 176 | 0.03 | 23.3 | NR | NR | M & F | 41.3 | NR | Poor |
| 28 | Noah et al | 2014 | 2012 | 2013 | Centre | Urban | Hospital-based | Liver Cancer Patients | CC | 33 | 40 | 82.5 | 41 | 8-77 | Beyond 15 | M & F | NR | NR | Moderate |
| 29 | Noah et al | 2014 | 2012 | 2013 | Centre | Urban | Hospital-based | General | CC | 25 | 40 | 62.5 | 40 | 11-82 | Beyond 15 | M & F | NR | RDT | Moderate |
| 30 | Abongwa et al | 2015 | 2015 | 2015 | Northwest | Urban & Rural | Hospital-based | Pregnant Women | CS | 19 | 301 | 6 | 25.7 | 13-44 | Beyond 15 | F | 0 | EIA | Moderate |
| 31 | Noubiap et al | 2015 | 2013 | 2014 | Far North | Rural | Hospital-based | Pregnant Women | CS | 33 | 325 | 10.2 | 24.4 | 15-40 | Beyond 15 | F | 0 | EIA | Poor |
| 32 | Shevell et al | 2015 | 2011 | 2011 | Southwest | Urban & Rural | Population-based | General | CS | 75 | 761 | 9.86 | 35.21 | 18-94 | Beyond 15 | M & F | 40.32 | RDT | Moderate |
| 33 | Loriette et al | 2015 | 2009 | 2013 | Far North | Rural | Hospital-based | Healthcare Workers | CS | 17 | 93 | 18 | NR | NR | NR | M & F | NR | RDT | High |
| 34 | Loriette et al | 2015 | 2009 | 2013 | Far North | Rural | Hospital-based | HIV-infected | CS | 143 | 995 | 14.4 | NR | NR | NR | M & F |  | RDT | High |
| 35 | Loriette et al | 2015 | 2009 | 2013 | Far North | Rural | Hospital-based | Blood Donors | CS | 393 | 2326 | 16.9 | NR | NR | NR | M & F |  | RDT | High |
| 36 | Loriette et al | 2015 | 2009 | 2013 | Far North | Rural | Hospital-based | Pregnant Women | CS | 2408 | 13208 | 18.2 | NR | NR | NR | F |  | RDT | High |
| 37 | Noah et al | 2015 | 2014 | 2014 | National | Urban | Population-based | General | CS | 568 | 4367 | 13.01 | 20.5 | 18-23 | Beyond 15 | M | 100 | RDT & ELISA | Poor |
| 38 | Noubiap et al | 2015 | 2000 | 2013 | Centre, Eastn South, Southwest, Northwest | Urban & Rural | NR | HIV-infected | RCS | 168 | 531 | 31.6 | NR | 14-NR | NR | M & F | 32 | ELISA | Moderate |
| 39 | Essomba et al | 2015 | 2014 | 2014 | Littoral | Urban | Hospital-based | Blood Donors | CS | 20 | 167 | 11.9 | 31.5 | 19-56 | Beyond 15 | M & F | 83.8 | RDT & ELISA | Poor |
| 40 | Mbopi-Kéou et al | 2015 | 2012 | 2012 | West | Urban & Rural | Population-based | General | CS | 41 | 982 | 4.1 | NR | 15-NR | Beyond 15 | M & F | 43.7 | NR | High |
| 41 | Abongwa et al | 2016 | 2014 | 2014 | Northwest | Urban & Rural | Hospital-based | Pregnant Women | CS | 20 | 260 | 7.7 | 29.42 | 17-44 | Beyond 15 | F | 0 | RDT | Moderate |
| 42 | Amougou et al | 2016 | 2013 | 2014 | Centre | Urba, | Hospital-based | Liver Cancer Patients | CC | 58 | 88 | 65.9 | 46 | 8-93 | Beyond 15 | M & F | 69.32 | ELISA | Moderate |
| 43 | Amougou et al | 2016 | 2013 | 2014 | Centre | Urban | Hospital-based | General | CC | 9 | 85 | 10.6 | 41.1 | 11-82 | NR | M & F | 69.4 | ELISA | Moderate |
| 44 | Tatsilong et al | 2016 | 2014 | 2014 | Centre | Urban | Hospital-based | Healthcare Workers | CS | 11 | 100 | 11 | 30.5 | NR | NR | M & F | 29 | RDT | Moderate |
| 45 | Salpini et al | 2016 | 2010 | 2012 | Centre | Urban | Hospital-based | HIV-infected | CS | 25 | 212 | 11.8 | NR | NR | NR | M & F | 27.4 | ELISA | High |
| 46 | Essome et al | 2016 | 2015 | 2015 | Centre | Urban | Hospital-based | General | CS | 8 | 182 | 4.39 | 33.76 | 18-48 | Beyond 15 | F | 0 | RDT | Poor |
| 47 | Dionne-Odom et al | 2016 | 2014 | 2014 | Littoral, Southwest | Urban & Rural | Hospital-based | Blood Donors | CS | 287 | 4225 | 6.8 | NR | NR | NR | M & F | NR | RDT | Moderate |
| 48 | Dionne-Odom et al | 2016 | 2014 | 2014 | Littoral, Southwest | Urban & Rural | Hospital-based | Pregnant Women | CS | 308 | 7069 | 4.4 | NR | NR | NR | F | 0 | RDT | Moderate |
| 49 | Enow-Tanjong et al | 2016 | 2010 | 2010 | Southwest | Urban & Rural | Hospital-based | Pregnant Women | CS | 40 | 406 | 9.9 | 26 | 15-47 | Beyond 15 | F | 0 | ELISA | Poor |
| 50 | Halle et al | 2016 | 2012 | 2013 | Littoral | Urban & Rural | Hospital-based | Heamodialysis Patients | CS | 6 | 97 | 6.2 | 51 | NR | NR | M & F | 66 | EIA | Moderate |
| 51 | Tagny et al | 2016 | 2015 | 2015 | Centre | Urban | Hospital-based | General | CS | 123 | 1596 | 7.7 | 29 | 16-59 | Beyond 15 | M & F | 82.3 | EIA | Moderate |
| 52 | Luma et al, 2016 | 2016 | 2014 | 2015 | Southwest | Urban & Rural | Hospital-based | HIV-infected | CS | 51 | 833 | 6.1 | 44 | 21-NR | Beyond 15 | M & F | 21.1 | RDT | Moderate |
| 53 | Mondinde Ikomey et al | 2016 | 2014 | 2014 | Southwest | Urban & Rural | Hospital-based | HIV-Pregnant Women | CS | 14 | 97 | 14.4 | 25.72 | 20-43 | Beyond 15 | F | 0 | ELISA | Poor |
| 54 | Ankouane et al | 2016 | 2013 | 2013 | Centre | Urban | Hospital-based | Blood Donors | RCS | 1137 | 9024 | 12.6 | 28 | NR | NR | M & F | 97 | RDT | Poor |
| 55 | Magoro et al | 2016 | NR | NR | Littoral, Southwest | Urban & Rural | Hospital-based | HIV-infected | CS | 116 | 455 | 25.5 | 33.8 | 1-68 | Beyond 15 | M & F | 74.6 | ELISA | Poor |
| 56 | Sone et al | 2017 | NR | NR | Centre | Urban | Hospital-based | Pregnant Women | CS | 23 | 298 | 7.7 | 26.3 | 15-42 | Beyond 15 | F | 0 | ELISA | Poor |
| 57 | Luma et al | 2017 | 2013 | 2013 | Northwest, Southwest | Urban | Hospital-based | Heamodialysis Patients | CS | 11 | 104 | 10.6 | 48 | NR | NR | M & F | 65.4 | EIA | Poor |
| 58 | Nansseu et al | 2017 | 2014 | 2014 | West | Rural | Population-based | General | CS | 20 | 443 | 4.5 | 45.3 | 1-92 | Beyond 15 | M & F | 28.9 | RDT | Moderate |
| 59 | Rodgers et al | 2017 | 2011 | 2015 | South | Rural | Population-based | General | CS | 1431 | 13695 | 10.45 | NR | 0-92 | NR | M & F | 34 | ELISA | High |
| 60 | Philemon et al | 2018 | 2014 | 2014 | Centre, Far North, East, Littoral, West | Urban & Rural | Hospital-based | General | CS | 22 | 405 | 5.43 | 39.4 | 3-88 | Beyond 15 | M & F | 34.3 | RDT | Moderate |
| 61 | Torimiro et al | 2018 | 2011 | 2015 | Centre | Urban | Hospital-based | Pregnant Women | CS | 64 | 1002 | 6.4 | 27.49 | 18-43 | Beyond 15 | F | 0 | ELISA | Moderate |
| 62 | Molu et al | 2018 | 2008 | 2014 | Centre | Rural | Hospital-based | HIV-infected | CS | 64 | 712 | 8.99 | 38.9 | 4-93 | Beyond 15 | M & F | NR | RDT | Moderate |
| 63 | Fouelifack et al | 2018 | 2016 | 2016 | Centre | Urban | Hospital-based | Pregnant Women | CS | 34 | 360 | 9.4 | 27.9 | 15-NR | Beyond 15 | F | 0 | RDT | Poor |
| 64 | Ndongo et al | 2018 | 2016 | 2016 | National | Urban & Rural | Hospital-based | Healthcare Workers | CS | 156 | 1790 | 8.7 | 34 | 15-NR | Beyond 15 | M & F | 35 | RDT | High |
| 65 | Pinho-Nascimento et al | 2018 | NR | NR | Adamawa | Rural | Population-based | General | CS | 54 | 401 | 13.46 | NR | 0-NR | NR | NR | NR | ELISA | Moderate |
| 66 | Kengne et al | 2018 | 2014 | 2014 | Centre | Urban | Hospital-based | Blood Donors | CS | 31 | 265 | 11.7 | 28 | 18-52 | Beyond 15 | M & F | 91.4 | ELISA | Poor |
| 67 | Fopa et al | 2018 | 2016 | 2017 | Centre | Urban | Hospital-based | Blood Donors | CS | 91 | 1162 | 7.8 | 29.24 | 18-65 | Beyond 15 | M & F | 86.2 | RDT | Moderate |
| 68 | Fopa et al | 2019 | NR | NR | Centre | Urban | Hospital-based | Blood Donors | CS | 91 | 1166 | 7.8 | NR | NR | NR | NR | NR | NR | Poor |
| 69 | Fokam et al | 2019 | 2017 | 2017 | Centre | Urban | Hospital-based | HIV-infected | CS | 2 | 83 | 2.4 | 8.7 | 0-15 | Under 15 | M & F | 45.8 | RDT & EIA | Moderate |
| 70 | Kalla et al | 2019 | 2018 | 2018 | NR | Urban & Rural | Population-based | General | CS | 104 | 1206 | 8.6 | 31.9 | 19-91 | Beyond 15 | M & F | 55.14 | ELISA | Poor |
| 71 | Mbock et al | 2019 | 2018 | 2018 | Centre | Urban | Hospital-based | Cancer Patients (not liver) | CS | 24 | 244 | 9.84 | 44.07 | 20-85 | Beyond 15 | M & F | 11.48 | RDT | Poor |
| 72 | Eyong et al | 2019 | 2014 | 2016 | Southwest | Urban & Rural | Hospital-based | Pregnant Women | CS | 175 | 2647 | 6.6 | 26.7 | 14-47 | Beyond 15 | F | 0 | NR | Poor |
| 73 | Tsague et al | 2019 | 2008 | 2014 | West | Urban & Rural | Hospital-based | General | CS | 498 | 5172 | 9.6 | NR | 0-101 | NR | M & F | 52.4 | ELISA | High |
| 74 | Ngoupa et al | 2019 | 2017 | 2017 | Centre, Littoral | Urban | NR | General | CS | 33 | 262 | 12.6 | NR | 2-62 | NR | M & F | 89 | RDT & ELISA | Poor |
| 75 | Torimiro et al | 2020 | 2019 | 2020 | Centre | NR | Hospital-based & Pre-School | General | CS | 2 | 300 | 0.7 | NR | 0-5 | Under 15 | M & F | 52 | ELISA | Moderate |
| 76 | Liegeois et al | 2020 | 2014 | 2014 | Centre, Littoral | Urban & Rural | Hospital-based | HIV-infected | CS | 159 | 1706 | 9.3 | NR | NR | NR | M & F | 26 | EIA | Moderate |
| 77 | Akazong et al | 2020 | 2017 | 2017 | Northwest | Urban & Rural | Hospital-based | Healthcare Workers | CS | 42 | 398 | 10.6 | NR | 16-65 | Beyond 15 | M & F | 31.7 | EIA | Poor |
| 78 | Besombes et al | 2020 | 2011 | 2011 | National | Urban & Rural | Population-based | General | CS | 1621 | 14150 | 11.9 | NR | NR-15 | Under 15 | M & F | NR | ELISA | High |
| 79 | Tadongfack et al | 2020 | 2018 | 2018 | West | Rural | Hospital-based | General | CS | 28 | 551 | 5.08 | NR | 2-NR | NR | M & F | 37.39 | ELISA | Moderate |
| 80 | Tazinkeng et al | 2021 | 2021 | 2021 | Southwest | Urban & Rural | Population-based | General | CS | 65 | 1144 | 5.7 | 30 | 15-NR | Beyond 15 | M & F | 34 | NR | Moderate |
| 81 | Ngueko et al | 2021 | 2018 | 2018 | Centre, Littoral, West | Urban & Rural | Hospital-based | Disable People | CS | 8 | 153 | 5.23 | 37 | NR | NR | M & F | 44.44 | RDT | Poor |
| 82 | Kowo et al | 2021 | 2019 | 2019 | East | Rural | Hospital-based | Refugees | CS | 75 | 970 | 7.7 | 29 | 2-87 | Beyond 15 | M & F | 0.43 | RDT | Moderate |
| 83 | Kowo et al | 2021 | 2018 | 2018 | Littoral | Urban | Prison-based | Prisoners | CS | 121 | 940 | 12.9 | 33.81 | 14-74 | Beyond 15 | M & F | 94 | RDT | Moderate |
| 84 | Samje et al | 2021 | 2019 | 2019 | Northwest | Urban & Rural | Hospital-based | Blood Donors | CS | 16 | 250 | 6.4 | NR | 18-57 | Beyond 15 | M & F | 70.4 | RDT | Poor |
| 85 | Nlinwe et al | 2021 | 2020 | 2020 | Northwest | Urban & Rural | Hospital-based | Pregnant Women | CS | 11 | 221 | 4.98 | NR | 15-49 | Beyond 15 | F | 0 | RDT | Moderate |
| 86 | Samje et al | 2021 | 2012 | 2017 | Northwest | Urban & Rural | Hospital-based | Blood Donors | CS | 571 | 12115 | 4.7 | NR | 19-64 | Beyond 15 | M & F | 85.2 | RDT | Poor |
| 87 | Tietcheu et al | 2022 | 2021 | 2021 | Adamawa | Urban & Rural | Hospital-based | General | CS | 50 | 225 | 22.22 | NR | 15-55 | Beyond 15 | M & F | 57.3 | RDT | Moderate |
| 88 | Mawouma et al | 2022 | 2020 | 2021 | Far North | Rural | Hospital-based | Pregnant Women | CS | 146 | 794 | 18.4 | NR | 15-48 | Beyond 15 | F | 0 | RDT | Moderate |
| 89 | Shimakawa et al | 2022 | 2009 | 2016 | Far North | Rural | Hospital-based | Pregnant Women | CS | 3901 | 22243 | 17.5 | 24 | 15-NR | Beyond 15 | F | 0 | ELISA | High |
| 90 | Tazinkeng et al | 2022 | 2021 | 2021 | Southwest | Rural | Hospital-based | General | CS | 65 | 1144 | 5.7 | NR | NR | NR | M & F | 33.6 | RDT | High |
| 91 | Bikoi et al | 2022 | 2018 | 2019 | Centre | Urban | Hospital-based | Cancer Patients (not liver) | CS | 5 | 91 | 5.5 | 56.5 | 19-78 | Beyond 15 | M & F | 69.23 | NR | Poor |
| 92 | Funeh et al | 2022 | 2015 | 2018 | Northwest | Rural | Hospital-based | General | CS | 70 | 1208 | 5.8 | 35.9 | 21-80 | Beyond 15 | M & F | 40.4 | RDT | Moderate |
| 93 | Voundi et al | 2023 | 2021 | 2021 | Centre | Urban | Hospital-based | Blood Donors | CS | 13 | 232 | 5.6 | NR | 18-60 | Beyond 15 | M & F | 72.5 | ELISA | Moderate |
| 94 | Nzechieu Evenge et al | 2023 | 2021 | 2021 | Southwest | Rural | Hospital-based | General | CS | 10 | 119 | 8.4 | NR | 13-69 | NR | M & F | 48.7 | RDT | High |
| 95 | Bouba et al | 2024 | 2020 | 2022 | Far North | Rural | Hospital-based | Blood Donors | CS | 183 | 1042 | 17.6 | NR | 20-NR | Beyond 15 | M & F | 59.7 | ELISA | Poor |
| 96 | National AIDS Control Committee | 2024 | 2023 | 2024 | National | Urban & Rural | Hospital-based | Pregnant Women | CS | 477 | 7283 | 6.5 | 26 | 15-49 | Beyond 15 | F | 0 | RDT | High |
| 97 | Ngomtcho et al | 2024 | 2022 | 2022 | Southwest | Rural | Hospital-based | Blood Donors | CS | 29 | 165 | 12.12 | 32.23 | 18-60 | Beyond 15 | M & F | 95.7 | RDT & ELISA | Moderate |
| 98 | Torimiro et al | 2024 | 2018 | 2022 | Centre | Urban & Rural | Hospital-based | Pregnant Women | CS | 115 | 1992 | 5.8 | NR | NR | NR | F | 0 | RDT & ELISA | Moderate |
| 99 | Ondigui et al | 2024 | 2019 | 2022 | Centre | Urban & Rural | Hospital-based | Pregnant Women | CS | 133 | 1992 | 6.7 | 27.53 | 15-45 | Beyond 15 | F | 0 | RDT & ELISA | High |
| 100 | Abessolo et al | 2025 | 2024 | 2024 | South | Rural | Hospital-based | HIV-infected | CS | 46 | 176 | 26.1 | 26.2 | 10-40 | Beyond 15 | M & F | 63.2 | RDT | Moderate |

**NR:** Not Reported; **CS:** Cross Sectional; **RCS:** Retrospective Cross Sectional; **EIA:** Enzyme Immune Assay; **ELISA:** Enzyme-Linked Immunosorbent Assay; **RDT:** rapid diagnostic test; **M:** Male; **F:** Female.

1.	Mbanya D, Binam F, Kaptue L. Transfusion outcome in a resource-limited setting of Cameroon: A five-year evaluation. Int J Infect Dis. 2001;5(2):70–3.

2.	Mbanya DN, Takam D, Ndumbe PM. Serological findings amongst first‐time blood donors in Yaoundé, Cameroon: is safe donation a reality or a myth? Transfus Med. 2003 Oct;13(5):267–73.

3.	Koanga Mogtomo ML, Louandji Fomekong S, Fotso Kuate H, Ngono Ngane A. Détection des agents infectieux dans les banques de sang de Douala (1995-2004). Cah Santé. 2009 Jan;19(1):3–8.

4.	Tagny CT, Diarra A, Yahaya R, Hakizimana M, Nguessan A, Mbensa G, et al. Characteristics of blood donors and donated blood in sub‐Saharan Francophone Africa. Transfusion (Paris). 2009 Aug;49(8):1592–9.

5.	Laurent C, Bourgeois A, Mpoudi‐Ngolé E, Kouanfack C, Ciaffi L, Nkoué N, et al. High rates of active hepatitis B and C co‐infections in HIV‐1 infected Cameroonian adults initiating antiretroviral therapy. HIV Med. 2010 Jan;11(1):85–9.

6.	Foupouapouognigni Y, Sadeuh Mba SA, À Betsem EB, Rousset D, Froment A, Gessain A, et al. Hepatitis B and C Virus Infections in the Three Pygmy Groups in Cameroon. J Clin Microbiol. 2011 Feb;49(2):737–40.

7.	Noah Noah D, Njouom R, Bonny A, Pirsou P, Meli J, Sida MB. HBs antigene prevalence in blood donors and the risk of transfusion of hepatitis b at the central hospital of yaounde, cameroon. Open J Gastroenterol. 2011;01(02):23–7.

8.	Kouanfack C, Aghokeng AF, Mondain AM, Bourgeois A, Kenfack A, Mpoudi-Ngolé E, et al. Lamivudine-Resistant HBV Infection in HIV-Positive Patients Receiving Antiretroviral Therapy in a Public Routine Clinic in Cameroon. Antivir Ther. 2012 Feb;17(2):321–6.

9.	Zoufaly A, Onyoh EF, Tih PM, Awasom CN, Feldt T. High prevalence of hepatitis B and syphilis co-infections among HIV patients initiating antiretroviral therapy in the north-west region of Cameroon. Int J STD AIDS. 2012 Jun;23(6):435–8.

10.	Fouelifack Ymele F, Keugoung B, Fouedjio JH, Kouam N, Mendibi S, Dongtsa Mabou J. High Rates of Hepatitis B and C and HIV Infections among Blood Donors in Cameroon: A Proposed Blood Screening Algorithm for Blood Donors in Resource-Limited Settings. J Blood Transfus. 2012 Oct 10;2012:1–7.

11.	Kfutwah AK, Tejiokem MC, Njouom R. A low proportion of HBeAg among HBsAg-positive pregnant women with known HIV status could suggest low perinatal transmission of HBV in Cameroon. Virol J. 2012 Dec;9(1):62.

12.	Ottop FM, Atashili J, Ndumbe PM. Impact of Antiretroviral Therapy on Glycemia and Transaminase Levels in Patients Living with HIV/AIDS in Limbe, Cameroon. J Int Assoc Provid AIDS Care JIAPAC. 2013 Feb;12(1):23–7.

13.	Mp H, Hn L. Prevalence of Hepatitis B Surface Antigen and Anti- HIV Antibodies among Patients on Maintenance Haemodialysis in Douala - Cameroon. 2013;14.

14.	Fomulu NJ, Morfaw FL, Torimiro JN, Nana P, Koh MV, William T. Prevalence, correlates and pattern of Hepatitis B among antenatal clinic attenders in Yaounde-Cameroon: is perinatal transmission of HBV neglected in Cameroon? BMC Pregnancy Childbirth. 2013 Dec;13(1):158.

15.	Noah Noah D, Ngaba GP, Eloumou SF, Assi C, Ngantchet E, Njoya O. Evaluation de l’état vaccinal contre l’hépatite B et portage de l’Ag HBs chez le personnel médical et paramédical de l’Hôpital Central de Yaoundé, Cameroun. Pan Afr Med J [Internet]. 2013 [cited 2025 Jul 6];16. Available from: http://www.panafrican-med-journal.com/content/article/16/111/full/

16.	Noubiap JJN, Joko WYA, Nansseu JRN, Tene UG, Siaka C. Sero-epidemiology of human immunodeficiency virus, hepatitis B and C viruses, and syphilis infections among first-time blood donors in Edéa, Cameroon. Int J Infect Dis. 2013 Oct;17(10):e832–7.

17.	Feldt T, Sarfo FS, Zoufaly A, Phillips RO, Burchard G, Van Lunzen J, et al. Hepatitis E virus infections in HIV-infected patients in Ghana and Cameroon. J Clin Virol. 2013 Sep;58(1):18–23.

18.	Rey-Cuille MA, Njouom R, Bekondi C, Seck A, Gody C, Bata P, et al. Hepatitis B Virus Exposure During Childhood in Cameroon, Central African Republic and Senegal After the Integration of HBV Vaccine in the Expanded Program on Immunization. Pediatr Infect Dis J. 2013 Oct;32(10):1110–5.

19.	Ducancelle A, Abgueguen P, Birguel J, Mansour W, Pivert A, Le Guillou-Guillemette H, et al. High Endemicity and Low Molecular Diversity of Hepatitis B Virus Infections in Pregnant Women in a Rural District of North Cameroon. Ahn SH, editor. PLoS ONE. 2013 Nov 12;8(11):e80346.

20.	Ngo Sack F, Noah Noah D, Zouhairatou H, Mbanya D. Portage de l’antigène HBs et des anticorps anti-VHC chez le drépanocytaire homozygote à l’Hôpital Central de Yaoundé. Pan Afr Med J [Internet]. 2013 [cited 2025 Jul 6];2. Available from: http://www.panafrican-med-journal.com/content/article/14/40/full/

21.	Fritzsche C, Becker F, Hemmer CJ, Riebold D, Klammt S, Hufert F, et al. Hepatitis B and C: neglected diseases among health care workers in Cameroon. Trans R Soc Trop Med Hyg. 2013 Mar;107(3):158–64.

22.	Eboumbou Moukoko CE, Ngo Sack F, Essangui Same EG, Mbangue M, Lehman LG. HIV, HBV, HCV and T. palliduminfections among blood donors and Transfusion-related complications among recipients at the Laquintinie hospital in Douala, Cameroon. BMC Hematol. 2014 Dec;14(1):5.

23.	Sobze MS, Donfack JH, Onohiol JF, Fokam J, Dongho GBD, Pete PSN, et al. Prevalence of HIV, HBV and chlamydia infections in Cameroonian University context: case of the University of Dschang, in the Western region. BMC Infect Dis. 2014 May;14(S2):P3, 1471-2334-14-S2-P3.

24.	Frambo AAB, Atashili J, Fon PN, Ndumbe PM. Prevalence of HBsAg and knowledge about hepatitis B in pregnancy in the Buea Health District, Cameroon: a cross-sectional study. BMC Res Notes. 2014 Dec;7(1):394.

25.	Noah Noah D, Nko’Ayissi G, Ankouane Andoulo F, Ndoye E, Njoum R. Présentation clinique, biologique et facteurs de risque du Carcinome hépatocellulaire : une étude Cas-Témoins à Yaoundé au Cameroun. Révue Med Pharm. 2014;4(2).

26.	Abongwa LE, Clara AM, Edouard NA, Ngum NH. Sero-Prevalence of Human Immunodeficiency Virus (HIV) and Hepatitis B Virus (HBV) Co-Infection among Pregnant Women Residing in Bamenda Health District, Cameroon. 2015;

27.	Noubiap JJN, Nansseu JRN, Ndoula ST, Bigna JJR, Jingi AM, Fokom-Domgue J. Prevalence, infectivity and correlates of hepatitis B virus infection among pregnant women in a rural district of the Far North Region of Cameroon. BMC Public Health. 2015 Dec;15(1):454.

28.	Shevell L, Meriki HD, Cho-Ngwa F, Fuller C. Epidemiology of human immunodeficiency virus-1 and hepatitis B virus co-infection and risk factors for acquiring these infections in the Fako division of Southwest Cameroon. BMC Public Health. 2015 Dec;15(1):1066.

29.	Loriette M, Birguel J, Damza R, Ratoua M, Karsikam S, Sobnangou JJ, et al. An experience of hepatitis B control in a rural area in Far North Cameroon. Médecine Santé Trop. 2015 Oct;25(4):422–7.

30.	Noah DN, Andoulo FA, Essoh BVM, Ayissi GN, Bagnaka SAFE, Sida MB. Prevalence of HBs Antigen, and HCV and HIV Antibodies in a Young Male Population in Cameroon. Open J Gastroenterol. 2015;05(12):185–90.

31.	Noubiap JJN, Aka PV, Nanfack AJ, Agyingi LA, Ngai JN, Nyambi PN. Hepatitis B and C Co-Infections in Some HIV-Positive Populations in Cameroon, West Central Africa: Analysis of Samples Collected Over More Than a Decade. Chemin IA, editor. PLOS ONE. 2015 Sep 15;10(9):e0137375.

32.	Essomba NE, Ngaba GP, Koum DCK, Momo L, Lehman L, Coppieters Y. Séroprévalence de l’infection au cytomégalovirus chez les donneurs de sang à l’hôpital de district de Bonassama Douala-Cameroun. 2015;

33.	Mbopi-Keou FX, Monthe IV, Mireille GC, Nguefack-Tsague G, Kamga HG, Noubom M, et al. Séroprévalence et facteurs associés au VIH et aux hépatites virales B et C dans la ville de Bafoussam au Cameroun. Pan Afr Med J [Internet]. 2015 [cited 2025 Jul 6];20.

34.	Abongwa LE, Kenneth P. Assessing prevalencee and risk factors of Hepatitis B surfacr antigen amongst pregnant women attending antenatal clinic in the Northwest Region of Cameroon. Eur J Res Med Sci. 2016;4(1):32–43.

35.	Amougou MA, Noah DN, Moundipa PF, Pineau P, Njouom R. A prominent role of Hepatitis D Virus in liver cancers documented in Central Africa. BMC Infect Dis. 2016 Dec;16(1):647.

36.	Tatsilong HOP, Noubiap JJN, Nansseu JRN, Aminde LN, Bigna JJR, Ndze VN, et al. Hepatitis B infection awareness, vaccine perceptions and uptake, and serological profile of a group of health care workers in Yaoundé, Cameroon. BMC Public Health. 2016 Dec;16(1):706.

37.	Salpini R, Fokam J, Ceccarelli L, Santoro MM, Nanfack A, Martin Sosso S, et al. High Burden of HBV-Infection and Atypical HBV Strains among HIV-infected Cameroonians. Curr HIV Res. 2016 Feb 1;14(2):165–71.

38.	Ngonde MC, Nsawir BJ, Nana RD, Molu P, Mohamadou M. Etude séro-epidémiologique de trois infections sexuellement transmissibles (Chlamydia Trachomatis, Hépatite B, Syphilis): cas de l’Hôpital de District de Nkoldongo à Yaoundé. Pan Afr Med J [Internet]. 2016 [cited 2025 Jul 6];25.

39.	Dionne-Odom J, Mbah R, Rembert NJ, Tancho S, Halle-Ekane GE, Enah C, et al. Hepatitis B, HIV, and Syphilis Seroprevalence in Pregnant Women and Blood Donors in Cameroon. Infect Dis Obstet Gynecol. 2016;2016:1–8.

40.	Tanjong RE, Teyim P, Kamga HL, Neba ES, Nkuo-Akenji T. Sero-prevalence of Human Immunodeficiency Virus and hepatitis viruses and their correlation with CD4 T-cell lymphocyte counts in pregnant women in the Buea Health District of Cameroon. Int J Biol Chem Sci. 2016 Aug 8;10(1):219.

41.	Halle Marie Patrice, Choukem Simeon-Pierre, Folefack Kaze François, Ashutantang Gloria, Tchamago Vanessa, Mboue-Djieka Yannick, et al. Hepatitis B, Hepatitis C, and Human Immune deficiency Virus Seroconversion Positivity Rates and Their Potential Risk Factors Among Patients on Maintenance Hemodialysis in Cameroon. Iran J Kidney Dis. 2016;10(5).

42.	Tagny CT, Ndoumba A, Laperche S, Murphy E, Mbanya D. Reducing risks of Transfusion‐transmitted infections in a resource‐limited hospital‐based blood bank: the case of the Yaoundé University Teaching Hospital, Cameroon. ISBT Sci Ser. 2016 Aug;11(2):82–7.

43.	Luma HN, Eloumou SAFB, Ekaney DSM, Lekpa FK, Donfack-Sontsa O, Ngahane BHM, et al. Sero-prevalence and Correlates of Hepatitis B and C Co-infection Among HIV-infected Individuals in Two Regional Hospitals in Cameroon. Open AIDS J. 2016 Nov 3;10(1):199–208.

44.	Mondinde Ikomey G. Evidence of Co and Triple Infections of Hepatitis B and C Amongst HIV Infected Pregnant Women in Buea, Cameroon. Sci J Public Health. 2016;4(2):127.

45.	Ankouane F, Noah Noah D, Atangana MM, Kamgaing Simo R, Guekam PR, Biwolé Sida M. Séroprévalence des virus des hépatites B et C, du VIH-1/2 et de la syphilis chez les donneurs de sang de l’hôpital central de Yaoundé, région du centre, Cameroun. Transfus Clin Biol. 2016 May;23(2):72–7.

46.	Magoro T, Gachara G, Mavhandu L, Lum E, Kimbi HK, Ndip RN, et al. Serologic and genotypic characterization of hepatitis B virus in HIV-1 infected patients from South West and Littoral Regions of Cameroon. Virol J. 2016 Dec;13(1):178.

47.	Etame Sone, PhD LH, Voufo, MSc RA, Dimodi, PhD HT, Kengne, PhD M, Gueguim, MSc C, Ngah, PhD N, et al. Prevalence and Identification of Serum Markers Associated with Vertical Transmission of Hepatitis B in Pregnant Women in Yaounde, Cameroon. Int J Matern Child Health AIDS IJMA. 2017 May 19;6(1):69–74.

48.	Luma HN, Halle MP, Fiacre SA, Azingala F, Kamdem F, Donfack-Sontsa O, et al. Seroprevalence of human immunodeficiency virus, hepatitis B and C viruses among haemodialysis patients in two newly opened centres in Cameroon. Pan Afr Med J [Internet]. 2017 [cited 2025 Jul 6];27.

49.	Nansseu JR, Mbogning DM, Monamele GC, Tamoh SF, Gonsu HK, Kouanfack C, et al. Sero-epidemiology of human immunodeficiency virus, hepatitis B virus and hepatitis C virus: a cross-sectional survey in a rural setting of the West region of Cameroon. Pan Afr Med J [Internet]. 2017 [cited 2025 Jul 6];28.

50.	Rodgers MA, Vallari AS, Harris B, Yamaguchi J, Holzmayer V, Forberg K, et al. Identification of rare HIV-1 Group N, HBV AE, and HTLV-3 strains in rural South Cameroon. Virology. 2017 Apr;504:141–51.

51.	Philemon EA. A Cross Sectional Study of the Impact of Human Immunodeficiency Virus, Hepatitis B Virus and Hepatitis C Virus on Rheumatoid Factor Production. Arch Rheumatol. 2018 Dec 6;33(4):402–7.

52.	Torimiro JN, Nanfack A, Takang W, Keou CK, Joyce AN, Njefi K, et al. Rates of HBV, HCV, HDV and HIV type 1 among pregnant women and HIV type 1 drug resistance-associated mutations in breastfeeding women on antiretroviral therapy. BMC Pregnancy Childbirth. 2018 Dec;18(1):504.

53.	Molu JP, Essome MCN, Monamele CG, Njouom R. Sero-prevalence of HBsAg in naive HIV-infected patients in a rural locality of Cameroon. BMC Res Notes. 2018 Dec;11(1):39.

54.	Fouelifack FY, Fouedjio JH, Fouogue JT, Fouelifa LD. Seroprevalences and Correlates of Hepatitis B and C Among Cameroonian Pregnant Women. Clin Med Insights Reprod Health. 2018 Jan 1;12:1179558118770671.

55.	Bilounga Ndongo C, Eteki L, Siedner M, Mbaye R, Chen J, Ntone R, et al. Prevalence and vaccination coverage of Hepatitis B among healthcare workers in Cameroon: A national seroprevalence survey. J Viral Hepat. 2018 Dec;25(12):1582–7.

56.	Pinho-Nascimento CA, Bratschi MW, Höfer R, Soares CC, Warryn L, Pečerska J, et al. Transmission of Hepatitis B and D Viruses in an African Rural Community. Whiteson KL, editor. mSystems. 2018 Oct 30;3(5):10.1128/msystems.00120-18.

57.	Kengne M, Wouado Tsata DC, Ndomgue T, Mbekem Nwobegahay J. Prevalence and risk factors of HTLV-1/2 and other blood borne infectious diseases among blood donors in Yaounde Central Hospital, Cameroon. Pan Afr Med J [Internet]. 2018 [cited 2025 Jul 6];30.

58.	Fopa D, Candotti D, Tagny CT, Doux C, Mbanya D, Murphy EL, et al. Occult hepatitis B infection among blood donors from Yaoundé, Cameroon. Blood Transfus. 2019 Sep 24;(Blood Transfusion-6 2019 (November-December)):403–8.

59.	Fopa, D, Tagny, C, Mbanya, Candotti D, Doux, C, Laperche, S, et al. Seroprevalence of viral infections in blood donors in Yaounde, Cameroon. Am J Trop Med Hyg. 2019;101(5):67.

60.	Joseph F, Rodrigue KW, Serges T, Salomon NP, Christian TN, Carlos TTM, et al. Hepatitis B infection and risk factors among children living with HIV in Yaounde, Cameroon: an integrated management. BMC Pediatr. 2019 Dec;19(1):366.

61.	Kalla GCM, Voundi EV, Guiadem R, Iii FA, Bélec L, Mbopi-Keou FX. Mass campaigns for HIV, HBV (HBsAg) and HCV screening by multiplex rapid diagnostic test in sub-Saharan Africa using mobile units: the game changer. Int J Infect Dis. 2019 Feb;79:107.

62.	Luc-Aime KS. Sero-Epidemiological Profile of Markers of Hepatitis B Viral Infection in Cancer Patients at the Yaounde General Hospital. 2019;

63.	Eyong EM, Yankam BM, Seraphine E, Ngwa CH, Nkfusai NC, Anye CS, et al. The prevalence of HBsAg, knowledge and practice of hepatitis B prevention among pregnant women in the Limbe and Muyuka Health Districts of the South West region of Cameroon: a three-year retrospective study. Pan Afr Med J [Internet]. 2019 [cited 2025 Jul 6];32.

64.	Kenfack Tsague M, Lambou Fotio A, Kamga Bomgning CL, Nguefack-Tsague G, Fopa F, Nguelefack TB. Prevalence of viral and non-viral hepatitis in Menoua Division, West Region, Cameroon: a retrospective hospital-based study. Pan Afr Med J [Internet]. 2019 [cited 2025 Jul 6];32.

65.	Ngoupa JB, Njukeng PA, Akwa EN, Kengne M, Tamoufe U, Goon DT, et al. Seroprevalence and associated risk factors for Hepatitis B virus infection among barbers and their clients in two cities in Cameroon. South Afr J Infect Dis. 2019 Jan 7;1–5.

66.	Torimiro JN, Teukeng C, Mafopa NG, Assene AN, Ondingui J, Awoumou PL, et al. High Vaccination Coverage against Hepatitis B Infection and Profile of Biomarkers of Infection in Children less than 60 Months of Age. 2020;21.

67.	for the EVOLCAM study group, Liégeois F, Boyer S, Eymard-Duvernay S, Carrieri P, Kouanfack C, et al. Hepatitis B testing, treatment, and virologic suppression in HIV-infected patients in Cameroon (ANRS 12288 EVOLCAM). BMC Infect Dis. 2020 Dec;20(1):49.

68.	Akazong W E, Tume C, Njouom R, Ayong L, Fondoh V, Kuiate JR. Knowledge, attitude and prevalence of hepatitis B virus among healthcare workers: a cross-sectional, hospital-based study in Bamenda Health District, NWR, Cameroon. BMJ Open. 2020 Mar;10(3):e031075.

69.	Besombes C, Njouom R, Paireau J, Lachenal G, Texier G, Tejiokem M, et al. The epidemiology of hepatitis delta virus infection in Cameroon. Gut. 2020 Jul;69(7):1294–300.

70.	Tadongfack TD, Nguepy Keubo FR, Bianke P. Hepatitis B infection in the rural area of Dschang, Cameroon: seroprevalence and associated factors. Pan Afr Med J [Internet]. 2020 [cited 2025 Jul 6];36.

71.	Tazingkeng N.N., Teuwafeu D.G., Asombang A.W., Ndip V. A., Bloom S.M., Nkhoma A., et al. Prevalence of and factors associated with hepatitis b and c in an urban health district in Cameroon, West Africa: A cross-sectional study. Am J Gastroenterol. 2021;116.

72.	Ngueko Y.A, Sobze M.S., Tsapi A.T., Biguioh R.M., Fokam J., Mansour B., et al. Mass screening for hepatitis B and C viruses in a population of persons with disabilities with and without HIV status in Cameroon. Ig E Sanità Pubblica. 2021;77(1):492–501.

73.	Kowo MP, Andoulo FA, Sizimboue DT, Ndjitoyap Ndam AW, Ngek LT, Kouanfack C, et al. Seroprevalence of hepatitis B and associated factors among inmates: a cross sectional study in the Douala New Bell Prison, Cameroon. Pan Afr Med J [Internet]. 2021 [cited 2025 Jul 6];38.

74.	Kowo MP, Frungwa CN, Njonnou SRS, Andoulo FA. Epidemiologic Patterns of HIV, Hepatitis B and C Virus Infections Among Refugees of the Mbile Camp in the East Region of Cameroon (Hepatitis and HIV among refugees).

75.	Samje M, Sop S, Tayou CT, Mbanya D. Knowledge, attitude and seropositivity of hepatitis B virus among blood donors in the Bamenda Regional Hospital Blood Bank, Cameroon. Pan Afr Med J [Internet]. 2021 [cited 2025 Jul 6];39.

76.	Nlinwe NO, Lungle D. Risk factors associated with hepatitis B virus infection among pregnant women attending the antenatal care unit of the Bamenda Regional Hospital. Public Health Pract. 2021 Nov;2:100160.

77.	Samje M, Fondoh VN, Nguefack-Tsague G, Kamalieuk LKJ, Mbanya D, Murphy EL, et al. Trends in serological markers of transfusion transmissible infections in blood donations at the Bamenda Hospital-based Blood Service, Cameroon. Transfus Clin Biol. 2021 Aug;28(3):228–33.

78.	Tietcheu BRG, Babai CN, Ngakou A. Seroprevalence, risk factors and impact of dengue fever/hepatitis B coinfection on liver function parameters in Cameroonian patients. Clin Exp Hepatol. 2022;8(2):161–9.

79.	Abdoul Rahamane Njigou Mawouma, Amadou Hapsatou Djoulatou, Eliane Ornella Komnang, Etienne Omolomo Kimessoukie. Facteurs associés à l’infection de l’hépatite B chez les femmes enceintes dans les formations sanitaires du district de santé de Mokolo/Région de l’Extrême-Nord Cameroun. PAMJ. 2022;41(61).

80.	Shimakawa Y, Veillon P, Birguel J, Pivert A, Sauvage V, Guillou-Guillemette HL, et al. Residual risk of mother-to-child transmission of hepatitis B virus infection despite timely birth-dose vaccination in Cameroon (ANRS 12303): a single-centre, longitudinal observational study. Lancet Glob Health. 2022 Apr;10(4):e521–9.

81.	Tazinkeng NN, Teuwafeu DG, Asombang AW, Agbor VN, Bloom SM, Nkhoma AN, et al. Factors associated with hepatitis B and C among adults in Buea, Cameroon: A community‐based cross‐sectional study. Liver Int. 2022 Nov;42(11):2396–402.

82.	Bikoï JN, Moni Ndedi EDF, Okobalemba EA, Mbaga DS, Mikangue CAM, Membangbi AE, et al. Seroprevalence of Epstein-Barr virus, hepatitis B virus, hepatitis C virus, and Human Immunodeficiency Virus (HIV) among patients with non-Hodgkin’s lymphoma at the Yaounde General Hospital Cameroon. Pan Afr Med J [Internet]. 2022 [cited 2025 Jul 6];42.

83.	Funeh CN, Ebasone PV, Chunga EM, Nkwawir F, Ajeh R, Barche B, et al. Seroprevalence of hepatitis B virus among people screened at a primary care hospital in Bamenda: a cross-sectional study.

84.	Voundi EV, Mintya AN, Bouopda R, Tagny CT, Mbanya D. Sécurité Transfusionnelle Infectieuse Pendant la Pandémie à COVID-19 : Evaluation de Quelques Marqueurs Sérologiques au Centre Hospitalier et Universitaire de Yaoundé, Cameroun. 2023;24.

85.	Nzechieu Evenge CN, Zeuko’o Menkem E, Ngounou E, Watching D, Nembu EN, Luma WS, et al. Prevalence of hepatitis B and associated factors in the Buea Regional Hospital, Cameroon. Heliyon. 2023 Jul;9(7):e17745.

86.	Bouba Gake, Avoula FL, Kouayet Fouayet Foyang YJ, Nukenine E. Prévalence de l’Hépatite B chez les Donneurs de Sang à Tokombéré (Extrême-Nord / Cameroun). Health Sci Dis. 2024;25(06):92–8.

87.	National AIDS Control Committee Report,. Minist Health Cameroon. 2024;

88.	Ngomtcho SCH, Njike Ngo Biyong O, Ekwere TA, Wandji Takemegni JM, Mbah H, Makamdoum Bogne SM, et al. Trends in immunological markers of transfusion transmissible infections among blood donors in Mamfe District Hospital, Southwest Cameroon. BMC Infect Dis. 2024 Apr 2;24(1):370.

89.	Torimiro JNE, Duri K, Goumkwa NM, Atah SM, Ndzie Ondigui JL, Lobe C, et al. Toward the elimination of hepatitis B: networking to promote the prevention of vertical transmission of hepatitis B virus through population-based interventions and multidisciplinary groups in Africa. Front Public Health. 2024 Apr 5;12:1283350.

90.	Ndzie Ondigui JL, Mafopa Goumkwa N, Lobe C, Wandji B, Awoumou P, Voussou Djivida P, et al. Prevalence and risk factors of transmission of hepatitis delta virus in pregnant women in the Center Region of Cameroon. Chemin I, editor. PLOS ONE. 2024 Jun 20;19(6):e0287491.

91.	Abessolo HA, Bakmano R, Liheb A, Moise A. Prévalence des Hépatites B et C dans un échantillon de Jeunes Personnes Vivant avec le VIH dans l’Arrondissement de Biwong Bane dans la Région du Sud-Cameroun. 2025;26.

92.	Hermine Abbessolo Abessolo, Raissa Bakmano, Armand Liheb, Adamou Moise. Prevalence of Hepatitis B and C in a Sample of Young Persons Living with HIV in the Biwong Bane District (South Region, Cameroon). Health Sci Dis. 2025;26(02):18–23.
